# Heuristic algorithms for best match graph editing

**DOI:** 10.1186/s13015-021-00196-3

**Published:** 2021-08-17

**Authors:** David Schaller, Manuela Geiß, Marc Hellmuth, Peter F. Stadler

**Affiliations:** 1 Max Planck Institute for Mathematics in the Sciences, Inselstraße 22, D-04109 Leipzig Leipzig, Germany; 2 Bioinformatics Group, Department of Computer Science, and Interdisciplinary Center for Bioinformatics, Universität Leipzig, Härtelstraße 16–18, D-04107 Leipzig, Germany; 3 Software Competence Center Hagenberg GmbH, Softwarepark 21, A-4232 Hagenberg, Austria; 4 Department of Mathematics, Faculty of Science, Stockholm University, SE-10691 Stockholm, Sweden; 5 Competence Center for Scalable Data Services and Solutions Dresden/Leipzig, Interdisciplinary Center for Bioinformatics, German Centre for Integrative Biodiversity Research (iDiv), and Leipzig Research Center for Civilization Diseases, Universität Leipzig, Augustusplatz 12, D-04107 Leipzig, Germany; 6 Department of Theoretical Chemistry, University of Vienna, Währinger Straße 17, A-1090 Vienna, Austria; 7 Facultad de Ciencias, Universidad National de Colombia, Sede Bogotá, Ciudad Universitaria, COL-111321 Bogotá, D.C. Colombia; 8 Santa Fe Institute, 1399 Hyde Park Rd., NM87501 Santa Fe, USA

**Keywords:** Arc modification problems, Heuristic algorithm, Consistent algorithm, NP-hardness

## Abstract

**Background:**

Best match graphs (BMGs) are a class of colored digraphs that naturally appear in mathematical phylogenetics as a representation of the pairwise most closely related genes among multiple species. An arc connects a gene *x* with a gene *y* from another species (vertex color) *Y* whenever it is one of the phylogenetically closest relatives of *x*. BMGs can be approximated with the help of similarity measures between gene sequences, albeit not without errors. Empirical estimates thus will usually violate the theoretical properties of BMGs. The corresponding graph editing problem can be used to guide error correction for best match data. Since the arc set modification problems for BMGs are NP-complete, efficient heuristics are needed if BMGs are to be used for the practical analysis of biological sequence data.

**Results:**

Since BMGs have a characterization in terms of consistency of a certain set of rooted triples (binary trees on three vertices) defined on the set of genes, we consider heuristics that operate on triple sets. As an alternative, we show that there is a close connection to a set partitioning problem that leads to a class of top-down recursive algorithms that are similar to Aho’s supertree algorithm and give rise to BMG editing algorithms that are consistent in the sense that they leave BMGs invariant. Extensive benchmarking shows that community detection algorithms for the partitioning steps perform best for BMG editing.

**Conclusion:**

Noisy BMG data can be corrected with sufficient accuracy and efficiency to make BMGs an attractive alternative to classical phylogenetic methods.

## Background

A wide range of tasks in computational biology start by determining, for a given “query gene” *x* in a species *A*, one or all genes *y* in another species *B* that are most similar to *x*. Conceptually, these *best matches* *y* of the query gene *x* are meant to approximate the set of genes in *B* that are evolutionary most closely related to *x*. Best matches can be identified by comparing evolutionary distances [1], which in turn are usually obtained from sequence alignments [2]. In practice, fast approximation algorithms such as blast and its successors are often used for this purpose [3, 4]. Even if sequence similarity is measured perfectly, deviations from a common molecular clock, i.e., differences in the evolutionary rates of different genes, cause discrepancies between *best hits* (most similar sequences) and *best matches* (evolutionary most closely related sequences), see [5] for a detailed discussion.

The idea of best matches in the sense of closest evolutionary relatedness pre-supposes an underlying tree *T* that describes the phylogenetic relationships among the genes, which correspond to the leaves of *T*, and a map $\sigma$ assigning to each gene *x* the species $\sigma (x)$ in which it resides. Given such a leaf-colored tree $(T,\sigma )$, the best match graph $\vec {G}(T,\sigma )$ has as its vertex set the leaves of *T*, i.e., the set of genes, and as (directed) arcs the best matches. The latter are defined as the pairs (*x*, *y*) for which the last common ancestor of *x* and *y* is at least as close to *x* as the last common ancestor of *x* and any other gene $y'$ from the same species $\sigma (y')=\sigma (y)$. Best match graphs (BMGs), i.e., digraphs that are derived from a leaf-labeled tree $(T,\sigma )$ in this manner (cf. Defs. 1 and 2 below), form a very restrictive class of colored digraphs [6, 7]. Empirically determined best hit data therefore will in general not satisfy the defining properties of BMGs. They can be corrected in part, however, by considering the problem of editing a given digraph to the closest BMG. We refer to this problem as *BMG editing* in line with the usual terminology to describe graph editing problems by the target class of graphs. Importantly, the input digraph will not be a BMG in general. In [8], it was shown that the arc modification problems for BMGs are NP-hard, but can be formulated as integer linear programs (ILPs) allowing practical solutions for small instances. However, in computational biology, applications to large gene families would be of particular interest, creating the need for faster, approximate solutions for BMG editing. Before embarking to develop software for a BMG-based analysis of large sequence data sets, we need to understand whether the editing problem for BMGs is tractable in practice with satisfactory accuracy and for interestingly large instances. The purpose of this contribution is to establish that this is indeed the case.

Motivated by both theoretical and practical considerations, we are mainly interested in heuristics that are consistent in the following sense: Let ${\mathbb {A}}$ be an algorithm that takes an arbitrary vertex-colored digraph $(\vec {G},\sigma )$ as input and outputs a BMG ${\mathbb {A}}(\vec {G},\sigma )$. Then ${\mathbb {A}}$ is consistent if ${\mathbb {A}}(\vec {G},\sigma )=(\vec {G},\sigma )$ whenever the input digraph $(\vec {G},\sigma )$ is a BMG. Such an algorithm can be obtained trivially by adding an initial check whether the input is a BMG or not. All algorithms described here, however, are designed such that their edit-operations leave BMGs unchanged.

**Fig. 1:**
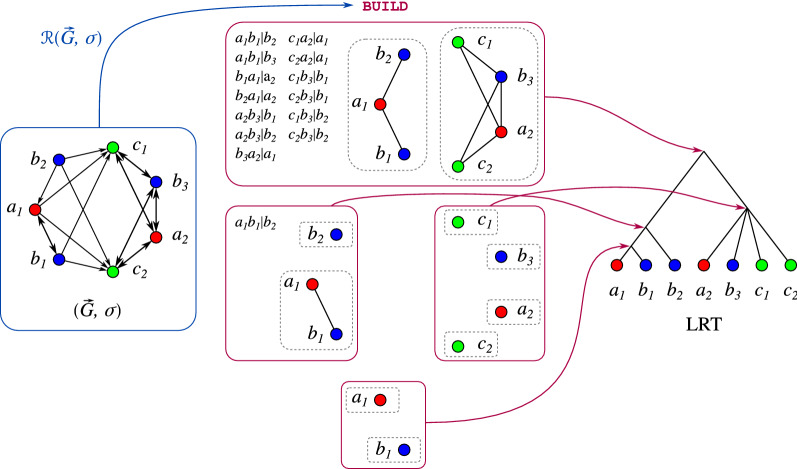
Construction of the LRT for a BMG $(\vec {G},\sigma )$ using the BUILD algorithm. Each recursion step (pink boxes) corresponds to a vertex of the resulting tree (trivial steps on single vertices are omitted in the drawing). The algorithm recurses on the connected components (gray dashed boxes) of the Aho graphs and the corresponding subsets of triples

BMGs can be characterized in terms of their set of so-called *informative (rooted) triples* ${\mathcal {R}}(\vec {G},\sigma )$ defined on the set of genes. These are binary trees with three leaves that can be easily extracted from a vertex-colored digraph (cf. Def. 6). In particular, ${\mathcal {R}}(\vec {G},\sigma )$ is consistent for a BMG $(\vec {G},\sigma )$, i.e., there is a common supertree for all triples in ${\mathcal {R}}(\vec {G},\sigma )$. A formal definition of triple consistency will be provided in the next section. In [7], it was shown that a vertex-colored digraph $(\vec {G}=(V,E),\sigma )$ is a BMG if and only if (a) the set of informative triples ${\mathcal {R}}(\vec {G},\sigma )$ is consistent and (b) the BMG $\vec {G}({{\hat{T}}}, \sigma )$ of the corresponding so-called Aho tree ${{\hat{T}}} {:}{=}{{\,\mathrm{Aho}\,}}({\mathcal {R}}(\vec {G},\sigma ),V)$ coincides with $(\vec {G},\sigma )$. In general, the Aho tree ${{\,\mathrm{Aho}\,}}({\mathcal {R}}, V)$ of a consistent set of triples ${\mathcal {R}}$ on a set *V* is a least resolved supertree of all the triples in ${\mathcal {R}}$. However, there are well-known examples for which the Aho tree is neither the unique least resolved supertree for ${\mathcal {R}}$ nor the tree with a minimal number of vertices [9]. For a BMG, on the other hand, ${{\hat{T}}} {:}{=}{{\,\mathrm{Aho}\,}}({\mathcal {R}}(\vec {G},\sigma ), V)$ is the *unique* least resolved tree (LRT) that explains $(\vec {G},\sigma )=\vec {G}({\hat{T}},\sigma )$ [6]. See Fig. 1 for an illustrative example of the construction of the LRT ${{\,\mathrm{Aho}\,}}({\mathcal {R}}(\vec {G},\sigma ),V)$ of a BMG $(\vec {G},\sigma )$ using the BUILD algorithm for supertree construction [10]. These close connections between recognizing BMGs and constructing supertrees suggest to adapt ideas from heuristic algorithms for triple consistency problems and supertree construction for BMG editing.

The simplest approach, therefore, is to extract a maximal consistent subset ${\mathcal {R}}^*$ from ${\mathcal {R}}(\vec {G},\sigma )$ and to use the BMG $\vec {G}({{\,\mathrm{Aho}\,}}({\mathcal {R}}^*,V),\sigma )$ as an approximation, see Alg. 1 below. A more detailed analysis of arcs in $(\vec {G},\sigma )$ that violate the property of being a BMG, however, will lead to a notion of “unsatisfiable relations” (UR), which can be used to count the arc modifications associated with a partition ${\mathcal {V}}$ of the vertex set *V* of $\vec {G}$. It also gives rise to a top-down algorithm in which the vertex set of $\vec {G}$ is recursively edited and partitioned. A large class of heuristics for BMG editing can be constructed depending on the construction of the partition ${\mathcal {V}}$ in each recursion step. We shall see that the arc edit sets in different steps of the recursion are disjoint. A main result of this contribution, Thm. 23, links the partitions ${\mathcal {V}}$ appearing in BMG editing algorithms to the auxiliary graphs appearing in the BUILD algorithm. This provides a guarantee that the BMG editing algorithms are consistent provided the choice of ${\mathcal {V}}$ is such that it does not enforce edits whenever an alternative partition with an empty UR is available. For BMGs, this is in particular the case for the partitions appearing in the BUILD algorithm. We proceed to show by reduction from Set Splitting that finding a partition with a minimal number of unsatisfiable relations is NP-hard.

The theoretical results are complemented by computational experiments on BMGs with randomly perturbed arc sets. To this end, we compare the arcs sets of the editing results with those of the perturbed digraphs and, since they are known in simulations, of the original BMGs. We focus on a comparison of different algorithms to construct the partitions ${\mathcal {V}}$. Somewhat surprisingly, we find that minimizing the cardinality of the UR alone is not the best approach, since this tends to produce very unbalanced partitions and thus requires a large number of steps in the recursions whose costs add up. Instead, certain types of clustering or community detection approaches that favor more balanced partitions tend to perform well in terms of the usual measures for digraph comparison such as the (absolute) symmetric difference, as well as recall, precision, and accuracy w.r.t. the arc sets. Finally, a brief analysis of the protein-coding genes of eleven Aquificales species suggests that our simulation results are a good representation of BMG editing in real-life applications.

## Theory

### Notation and Preliminaries

*Partitions.*${\mathcal {V}}=\{V_1,V_2,\dots ,V_k\}$ is a partition of a set *V* if (i) $V_i\ne \emptyset$, (ii) $\bigcup _{i=1}^k V_i = V$ and (iii) $V_i\cap V_j=\emptyset$ for $i\ne j$. A partition is non-trivial if $|{\mathcal {V}}|\ge 2$. Consider two partitions ${\mathcal {V}}=\{V_1,\dots ,V_k\}$ and ${\mathcal {V}}'=\{V'_1,\dots ,V'_l\}$ of *V*. If for every $1\le j'\le l$ there is a *j* such that $V'_{j'}\subseteq V_j$, i.e., if every set in ${\mathcal {V}}'$ is completely contained in a set in ${\mathcal {V}}$, then ${\mathcal {V}}'$ is a *refinement* of ${\mathcal {V}}$, and ${\mathcal {V}}$ is a *coarse-graining* of ${\mathcal {V}}'$.

*Graphs.* Mostly, we consider simple directed graphs (digraphs) $\vec {G}=(V,E)$ with vertex set *V* and arc set $E\subseteq V\times V \setminus \{(v,v)\mid v\in V\}$. We will frequently write $V(\vec {G})$ and $E(\vec {G})$ to explicitly refer to the graph $\vec {G}$. For a vertex $x\in V$, we say that (*y*, *x*) is an *in-arc* and (*x*, *z*) is an *out-arc*. The subgraph induced by a subset $W\subseteq V$ is denoted by $\vec {G}[W]$. Undirected graphs can be identified with symmetric digraphs, i.e., the undirected graph *G* underlying a digraph $\vec {G}$ is obtained by dropping the direction of all arcs, or by symmetrizing the digraph, i.e., adding the arc (*y*, *x*) to $E(\vec {G})$ for every arc $(x,y)\in E(\vec {G})$. When referring to an undirected graph *G*, we write *xy* for $(x,y),(y,x)\in E(G)$ and call *xy* an *edge*. The (weakly) connected components of $\vec {G}$ are the maximal connected subgraphs of the undirected graph underlying $\vec {G}$ or, equivalently, the maximal strongly connected components of the symmetrized digraph. Whenever the context is clear, we will also refer to the partition of *V* formed by the vertex sets of the maximal connected subgraphs as the set of connected components.

A vertex coloring is a map $\sigma :V\rightarrow S$, where *S* is a non-empty set of colors. A vertex coloring of $\vec {G}$ is *proper* if $\sigma (x)\ne \sigma (y)$ whenever $(x,y)\in E(\vec {G})$. We write $(\vec {G},\sigma )$ for a vertex-colored digraph and denote by *V*[*r*] the subset of vertices of a graph $(\vec {G}=(V,E),\sigma )$ that have color *r*. Moreover, we define $\sigma (W){:}{=}\{\sigma (x) \mid x\in W\}$ for the subset of colors present in a set $W\subseteq V$.

We write *N*(*x*) for the set of out-neighbors of a vertex $x\in V(\vec {G})$ and $N^-(x)$ for the set of in-neighbors of *x*. A digraph $\vec {G}$ is called *sink-free* if $N(x)\ne \emptyset$ holds for all $x\in V(\vec {G})$. We write $A{{\,\mathrm{\triangle }\,}}B {:}{=}(A\setminus B)\cup (B\setminus A)$ for the symmetric difference of two sets *A* and *B*, and define, for a digraph $\vec {G}=(V,E)$ and arc set $F\subseteq (V\times V)\setminus \{(v,v)\mid v\in V\}$, the digraph $\vec {G}{{\,\mathrm{\triangle }\,}}F{:}{=}(V, E{{\,\mathrm{\triangle }\,}}F)$. Analogously, we write $\vec {G}+ F{:}{=}(V, E\cup F)$ and $\vec {G}- F{:}{=}(V, E\setminus F)$.

*Phylogenetic trees.* Consider an undirected, rooted tree *T* with leaf set $L(T)\subseteq V(T)$ and root $\rho _T\in V(T)$. Its inner vertices are given by the set $V^0(T) = V(T) \setminus L(T)$. The *ancestor order* on *V*(*T*) is defined such that $u\preceq _T v$ if *v* lies on the unique path from *u* to the root $\rho _T$, i.e., if *v* is an ancestor of *v*. For brevity we set $u \prec _T v$ if $u \preceq _{T} v$ and $u \ne v$. If *xy* is an edge in *T* such that $y \prec _{T} x$, then *x* is the *parent* of *y* and *y* the *child* of *x*. The set of children of a vertex $x\in V(T)$ is denoted by $\mathsf {child}_T(x)$. A tree is *phylogenetic* if all of its inner vertices have at least two children. All trees considered in this contribution will be phylogenetic. For a non-empty subset $A\subseteq V(T)$, we define ${{\,\mathrm{lca}\,}}_T(A)$, the *last common ancestor of A*, to be the unique $\preceq _T$-minimal vertex of *T* that is an ancestor of every $u\in A$. Following e.g. [11], we denote by $T_{L'}$ the *restriction* of *T* to a subset $L'\subseteq L(T)$, i.e. $T_{L'}$ is obtained by identifying the (unique) minimal subtree of *T* that connects all leaves in $L'$, and suppressing all vertices with degree two except possibly the root $\rho _{T_{L'}}={{\,\mathrm{lca}\,}}_T(L')$. We say that *T* *displays* or *is a refinement of* a tree $T'$, in symbols $T'\le T$, if $T'$ can be obtained from a restriction $T_{L'}$ of *T* by a series of inner edge contractions. $(T,\sigma )$ is a leaf-colored tree if $\sigma : L(T)\rightarrow S$ is a map from the leaves of *T* into a non-empty set of colors. We say that $(T',\sigma ')$ is displayed by $(T,\sigma )$ if $T'\le T$ and $\sigma (v)=\sigma '(v)$ for all $v\in L(T')$.

*Rooted triples.* A *(rooted) triple* is a tree on three leaves and with two inner vertices, and thus, it has a topology as the tree in Fig. 3(D). We write *xy*|*z* for the triple on the leaves *x*, *y* and *z* if the path from *x* to *y* does not intersect the path from *z* to the root in *T*, i.e., if ${{\,\mathrm{lca}\,}}_T(x,y)\prec _T {{\,\mathrm{lca}\,}}_T(x,z)={{\,\mathrm{lca}\,}}_T(y,z)$. In this case we say that *T* displays *xy*|*z*. We write ${\mathcal {R}}_{|L'} {:}{=}\left\{ xy|z \in {\mathcal {R}} \,:x,y,z\in L' \right\}$ for the restriction of a triple set ${\mathcal {R}}$ to a set $L'$ of leaves. A set ${\mathcal {R}}$ of triples is *consistent* if there is a tree *T* with leaf set $L:=\bigcup _{T'\in {\mathcal {R}}} L(T')$ that displays every triple in ${\mathcal {R}}$. The polynomial-time algorithm BUILD decides for every triple set ${\mathcal {R}}$ whether it is consistent, and if so, constructs a particular tree, the *Aho tree* ${{\,\mathrm{Aho}\,}}({\mathcal {R}}, L)$, that displays every triple in ${\mathcal {R}}$ [10]. The algorithm relies on the construction of an (undirected) auxiliary graph, the *Aho graph*, for a given triple set ${\mathcal {R}}$ on a set of leaves *L*. This graph, denoted by $[{\mathcal {R}}, L]$, contains an edge *xy* if and only if $xy|z\in {\mathcal {R}}$ for some $z\in L$.

### Best match graphs

Best matches formalize the notion of the evolutionarily closest relative(s) of a gene *x* in another species. Relatedness in this context is thought of as a phylogenetic concept and thus expressed in terms of last common ancestors in the gene tree *T* that describes the evolutionary relationships among a family of genes.

#### Definition 1

Let $(T,\sigma )$ be a leaf-colored tree. A leaf $y\in L(T)$ is a *best match* of the leaf $x\in L(T)$ if $\sigma (x)\ne \sigma (y)$ and ${{\,\mathrm{lca}\,}}(x,y)\preceq _T {{\,\mathrm{lca}\,}}(x,y')$ holds for all leaves $y'$ of color $\sigma (y')=\sigma (y)$.

**Fig. 2:**
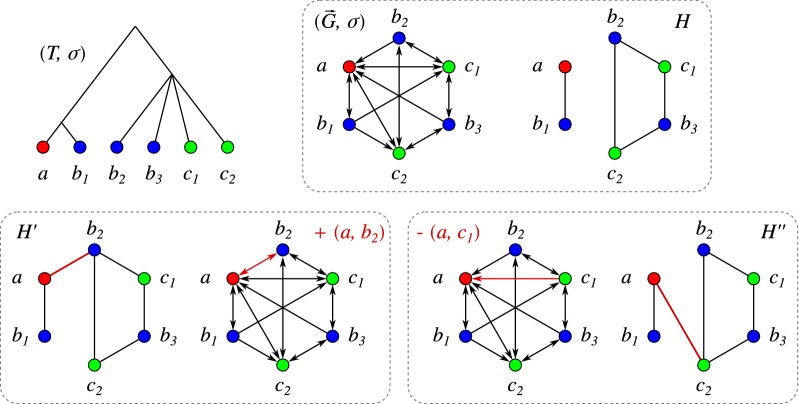
Both arc insertions and deletions into a BMG $(\vec {G}=(V,E),\sigma )$ can introduce inconsistencies into the set of informative triples. Top row: Leaf-colored tree $(T,\sigma )$ explaining the BMG $(\vec {G},\sigma )$. Its set of informative triples is ${\mathcal {R}}(\vec {G},\sigma )=\{ab_1|b_2,\, ab_1|b_3,\, c_1b_2|b_1,\, c_1b_3|b_1,\, c_2b_2|b_1,\, c_2b_3|b_1\}$ giving the Aho graph $H=[{\mathcal {R}}(\vec {G},\sigma ), V]$. Bottom left: Insertion of the arc $(a, b_2)$ creates a new informative triple $ab_2|b_3$ ($ab_1|b_2$ gets lost) resulting in a connected Aho graph $H'$. Bottom right: Deletion of the arc $(a, c_1)$ creates a new triple $ac_2|c_1$ resulting in a connected Aho graph $H''$

As a consequence, best matches in a pair of species in general form a many-to-many relationship and are not necessarily symmetric. Given $(T,\sigma )$, the digraph $\vec {G}(T,\sigma ) = (V,E)$ with vertex set $V=L(T)$, vertex-coloring $\sigma$, and with arcs $(x,y)\in E$ if and only if *y* is a best match of *x* w.r.t. $(T,\sigma )$ is called the *best match graph* (BMG) of $(T,\sigma )$ [6], see Fig. 2 for an illustrative example.

#### Definition 2

An arbitrary vertex-colored digraph $(\vec {G},\sigma )$ is a *best match graph (BMG)* if there exists a leaf-colored tree $(T,\sigma )$ such that $(\vec {G},\sigma ) = \vec {G}(T,\sigma )$. In this case, we say that $(T,\sigma )$ *explains* $(\vec {G},\sigma )$.

We say that $(\vec {G}=(V,E),\sigma )$ is an $\ell$-BMG if $|\sigma (V)|=\ell$. By construction, there is at least one best match of *x* for every color $s\in \sigma (V)\setminus \{\sigma (x)\}$:

#### Observation 3

*For every vertexxand every color*$s\ne \sigma (x)$*in a BMG*$(\vec {G},\sigma )$*there is some vertex*$y\in N(x)$*with*$\sigma (y)=s$. *Equivalently, the subgraph induced by every pair of colors is sink-free*.

In particular, therefore, BMGs are sink-free whenever they contain at least two colors. We formalize this basic property of BMGs for colored digraphs in general:

#### Definition 4

Let $(\vec {G}=(V,E),\sigma )$ be a colored digraph. The coloring $\sigma$ is *sink-free* if it is proper and, for every vertex $x\in V$ and every color $s\ne \sigma (x)$ in $\sigma (V)$, there is a vertex $y\in N(x)$ with $\sigma (y)=s$. A digraph with a sink-free coloring is called *sf-colored*.

Given a tree *T* and an edge *e*, we denote by $T_e$ the tree obtained from *T* by contracting the edge *e*. We call an edge in $(T,\sigma )$ *redundant (w.r.t.* $(\vec {G},\sigma )$) if both $(T,\sigma )$ and $(T_e,\sigma )$ explain $(\vec {G},\sigma )$.

#### Definition 5

A tree $(T,\sigma )$ is *least resolved* for a BMG $(\vec {G},\sigma )$ if (i) it explains $(\vec {G},\sigma )$ and (ii) it does not contain any redundant edges w.r.t. $(\vec {G},\sigma )$.

By [6, Thm. 8], every BMG has a unique least resolved tree (LRT). Moreover, a characterization of BMGs was given in [6] that makes use of a set of informative triples, which can be defined compactly as follows [12]:

#### Definition 6

Let $(\vec {G},\sigma )$ be a vertex-colored digraph. Then the set of *informative triples* is

$$\begin{aligned} {} & {\mathcal{R}} ({\vec{G}},\sigma ) \, {:=} \, \{ ab|b' :\sigma (a)\ne \sigma (b)=\sigma (b'), \\ &\quad (a,b)\in E({\vec{G}}), \ {\text{and}} \ (a,b')\notin E({\vec{G}}) \}, \end{aligned}$$

and the set of *forbidden triples* is

$$\begin{aligned} {} & {\mathcal{F}}({\vec{G}},\sigma ) \, {:=} \,\{ ab|b' :\sigma (a)\ne \sigma (b)=\sigma (b'), \\ &\quad b\ne b',\ {\text{and}} \ (a,b),(a,b')\in E({\vec{G}}) \}. \end{aligned}$$

For the subclass of BMGs that can be explained by binary trees, we will furthermore need

$$\begin{aligned} {} & \mathop{{\mathcal{R}}^{\text {B}}} ({\vec{G}},\sigma ) \, {:=} \, {\mathcal{R}} ({\vec{G}},\sigma ) \cup \\&\quad \{ bb'|a:ab|b'\in {\mathcal{F}}({\vec{G}},\sigma ), \sigma (b)=\sigma (b')\}. \end{aligned}$$

By definition, $a,b,b'$ must be pairwise distinct whenever $ab|b'\in {\mathcal {R}}(\vec {G},\sigma )$, $ab|b'\in {\mathcal {F}}(\vec {G},\sigma )$, or $ab|b'\in \mathop {{\mathcal {R}}^{\text {B}}}(\vec {G},\sigma )$.

We extend the notion of consistency to pairs of triple sets in

#### Definition 7

Let ${\mathcal {R}}$ and ${\mathcal {F}}$ be sets of triples. The pair $({\mathcal {R}},{\mathcal {F}})$ is called *consistent* if there is a tree *T* that displays all triples in ${\mathcal {R}}$ but none of the triples in ${\mathcal {F}}$. In this case, we also say that *T* *agrees with* $({\mathcal {R}},{\mathcal {F}})$.

It can be decided in polynomial time whether such a pair $({\mathcal {R}},{\mathcal {F}})$ is consistent using the algorithm MTT, which was named after the corresponding *mixed triplets problem restricted to trees* and described in [13].

We continue with two simple observations concerning the restriction of triple sets. Since informative and forbidden triples *xy*|*z* are only defined by the presence and absence of arcs in the subgraph of $\vec {G}$ induced by $\{x,y,z\}$, this leads to the following

#### Observation 8

[14] *Let* $(\vec {G},\sigma )$ *be a vertex-colored digraph and* $V'\subseteq V(\vec {G})$. *Then* $R(\vec {G},\sigma )_{|V'}=R(\vec {G}[V'],\sigma _{|V'})$ *holds for every* $R\in \{{\mathcal {R}},{\mathcal {F}}, \mathop {{\mathcal {R}}^{\text {B}}}\}$.

Moreover, any pair of triples $({\mathcal {R}}',{\mathcal {F}}')$ such that ${\mathcal {R}}'\subseteq {\mathcal {R}}$ and ${\mathcal {F}}'\subseteq {\mathcal {F}}$ for a consistent pair $({\mathcal {R}},{\mathcal {F}})$ remains consistent since any tree that agrees with $({\mathcal {R}},{\mathcal {F}})$ clearly displays all triples in ${\mathcal {R}}'$ and none of the triples in ${\mathcal {F}}'$. Hence, we have

#### Observation 9

*Let*${\mathcal {R}}'\subseteq {\mathcal {R}}$*and*${\mathcal {F}}'\subseteq {\mathcal {F}}$*for a consistent pair oftriple sets*$({\mathcal {R}},{\mathcal {F}})$. *Then*$({\mathcal {R}}',{\mathcal {F}}')$*is consistent.*

We summarize two characterizations of BMGs given in [7, Thm. 15] and [8, Lemma 3.4 and Thm. 3.5] in the following

#### Proposition 10

*Let*$(\vec {G},\sigma )$*be a properly colored digraph with vertex setL*. *Then the following three statements are equivalent:*

1. $$(\vec {G},\sigma )$$ is a BMG.
2. ${\mathcal {R}}(\vec {G},\sigma )$ is consistent and $\vec {G}({{\,\mathrm{Aho}\,}}({\mathcal {R}}(\vec {G},\sigma ),L), \sigma ) = (\vec {G},\sigma )$.
3. $(\vec {G},\sigma )$ is sf-colored and $({\mathcal {R}}(\vec {G},\sigma ),{\mathcal {F}}(\vec {G},\sigma ))$ is consistent.

In this case, $({{\,\mathrm{Aho}\,}}({\mathcal {R}}(\vec {G},\sigma ),L),\sigma )$ is the unique LRT for $(\vec {G},\sigma )$, and a leaf-colored tree $(T,\sigma )$ on *L* explains $(\vec {G},\sigma )$ if and only if it agrees with $({\mathcal {R}}(\vec {G},\sigma ), {\mathcal {F}}(\vec {G},\sigma ))$.

Prop. 10 states that the set of informative triples ${\mathcal {R}}(\vec {G},\sigma )$ of a BMG $(\vec {G},\sigma )$ is consistent. Therefore, it can be used to construct its LRT by means of the BUILD algorithm, see Fig. 1 for an example.

It is important to note that both arc insertions and deletions may lead to creation and loss of informative triples. In particular, when starting from a BMG, both types of modifications have the potential to make the triple set inconsistent as the example in Fig. 2 shows. This is indeed often the case even for moderate disturbances of a BMG as we shall see later.

We expect that empirically estimated best match relations will typically contain errors that correspond to both arc insertions and deletions w.r.t. the unknown underlying “true” best match graph. This motivates the problem of editing a given vertex-colored digraph to a BMG:

#### Problem 1

($$\ell $$-BMG Editing)

**Table:** 

*Input:*	A properly $\ell$-colored digraph
	$(\vec {G}=(V,E),\sigma )$ and an integer *k*.
*Question:*	Is there a subset
	$F\subseteq V\times V \setminus \{(v,v)\mid v\in V\}$ such that
	$|F|\le k$ and $(\vec {G}{{\,\mathrm{\triangle }\,}}F,\sigma )$ is an $\ell$-BMG?

Natural variants are $\ell$-BMG Completion and $\ell$-BMG Deletion where $\vec {G}{{\,\mathrm{\triangle }\,}}F$ is replaced by $\vec {G}+F$ and $\vec {G}-F$, respectively, i.e., only addition or deletion of arcs is allowed. Both $\ell$-BMG Editing and its variants are NP-complete [8].

The heuristic algorithms considered in this contribution can be thought of as maps ${\mathbb {A}}$ on the set of finite vertex-colored digraphs such that ${\mathbb {A}}(\vec {G},\sigma )$ is a BMG for every vertex-colored input digraph $(\vec {G},\sigma )$. In particular, the following property of such algorithms is desirable:

#### Definition 11

A (BMG-editing) algorithm is *consistent* if ${\mathbb {A}}(\vec {G},\sigma )=(\vec {G},\sigma )$ whenever $(\vec {G},\sigma )$ is a BMG.

### A simple, triple-based heuristic

The triple-based characterization summarized by Prop. 10 suggests a simple heuristic for BMG editing that relies on replacing the consistency checks for triple sets by the extraction of maximal sets of consistent triples (see Alg. 1). Both MaxRTC, the problem of extracting from a given set ${\mathcal {R}}$ of rooted triples a maximum-size consistent subset, and MinRTI, the problem of finding a minimum-size subset ${\mathcal {I}}$ such that ${\mathcal {R}}\setminus {\mathcal {I}}$ is consistent, are NP-hard [15]. Furthermore, MaxRTC is APX-hard and MinRTI is $\Omega (\ln n)$-inapproximable [16]. However, because of their practical importance in phylogenetics, a large number of practically useful heuristics have been devised, see e.g. [17–19].

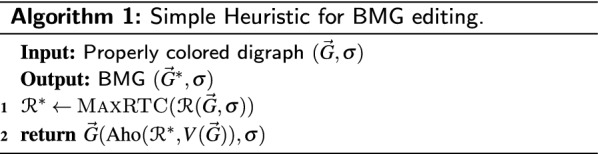



As a consequence of Prop. 10, Alg. 1 is consistent, i.e., $(\vec {G}^*,\sigma )=(\vec {G},\sigma )$ if and only if the input digraph $(G,\sigma )$ is a BMG, if a consistent heuristic is employed to solve MaxRTG/MinRTI, i.e., if consistent triple sets remain unchanged by the method approximating MaxRTG / MinRTI.

**Fig. 3:**
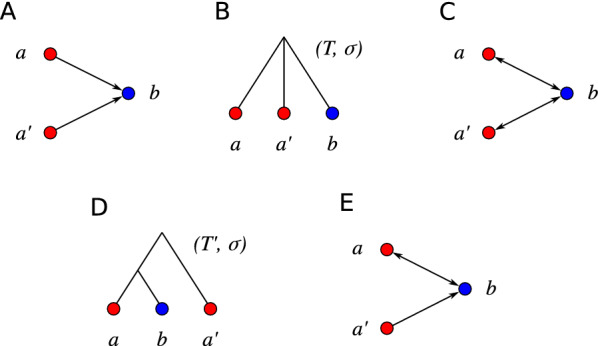
Example for a digraph (**A**) where Alg. 1 does not lead to an optimal BMG editing. The set ${\mathcal {R}}(\vec {G},\sigma )$ is empty and thus consistent. **B** The tree $T={{\,\mathrm{Aho}\,}}({\mathcal {R}}(\vec {G},\sigma ), V(\vec {G}))$ and (**C**) its corresponding BMG. The two arcs (*b*, *a*) and $(b,a')$ have been inserted. **D** A tree $(T',\sigma )$ and (E) its corresponding BMG $\vec {G}(T',\sigma )$ in which only the arc (*b*, *a*) has been inserted

The heuristic Alg. 1 is not always optimal, even if MaxRTC/MinRTI is solved optimally. Fig. 3 shows an unconnected 2-colored digraph $(\vec {G},\sigma )$ on three vertices that is not a BMG and does not contain informative triples. The BMG $(\vec {G}^*,\sigma )$ produced by Alg. 1 introduces two arcs into $(\vec {G},\sigma )$. However, $(\vec {G},\sigma )$ can also be edited to a BMG by inserting only one arc.

A simple improvement is to start by enforcing obvious arcs: If *v* is the only vertex with color $\sigma (v)$, then by definition there must be an arc (*x*, *v*) for every vertex $x\ne v$. The computation then starts from the sets of informative triples of the modified digraph. We shall see below that these are the only arcs that can safely be added to $\vec {G}$ without other additional knowledge or constraints (cf. Thm. 19 below).

### Locally optimal splits

Finding an optimal BMG editing of a digraph $(\vec {G}=(V,E),\sigma )$ is equivalent to finding a tree $(T,\sigma )$ on *V* that minimizes the cardinality of

1 $$\begin{aligned} U(\vec {G},T) {:}{=}\{ (x,y)\in V\times V \mid \ &(x,y)\in E \text { and }\\ &(x,y)\notin E(\vec {G}(T,\sigma )) \, \text {, or} \\& (x,y)\notin E \text { and }\\& (x,y)\in E(\vec {G}(T,\sigma )) \}. \end{aligned}$$

Clearly, $U(\vec {G},T) =\emptyset$ implies that $(\vec {G},\sigma ) = \vec {G}(T,\sigma )$ is a BMG. However, finding a tree $(T,\sigma )$ that minimizes $|U(\vec {G},T)|$ is intractable (unless $P=NP$) since $\ell$-BMG Editing, Problem 1 above, is NP-complete [8].

We may ask, nevertheless, if trees $(T,\sigma )$ on *V* contain information about arcs and non-arcs in $(\vec {G},\sigma )$ that are “unambiguously false” in the sense that they are contained in every edit set that converts $(\vec {G},\sigma )$ into a BMG. Denote by ${\mathcal {T}}_V$ the set of all phylogenetic trees on *V*. The set of these “unambiguously false” (non-)arcs can then be expressed as

2 $$\begin{aligned} U^*(\vec {G}){:}{=}\bigcap _{T\in {\mathcal {T}}_V} U(\vec {G},T). \end{aligned}$$

Since there are in general exponentially many trees on *V* and thus, the problem of determining $U^*(\vec {G})$ seems to be quite challenging at first glance. We shall see in Thm. 19, however, that $U^*(\vec {G})$ can be computed efficiently. We start with a conceptually simpler construction, and consider the set of trees ${\mathcal {T}}({\mathcal {V}})\subseteq {\mathcal {T}}_V$ for which the set of leaf sets of the children of the root equals the partition ${\mathcal {V}}$. In other words, given ${\mathcal {V}}=\{V_1,\dots ,V_k\}$, then the root $\rho _T$ of every $T\in {\mathcal {T}}({\mathcal {V}})$ has exactly *k* children $v_1,\dots ,v_k$ such that $V_i=L(T(v_i))$ for all $1\le i\le k$.

#### Definition 12

Let $(\vec {G}=(V,E),\sigma )$ be a properly vertex-colored digraph and ${\mathcal {V}}$ a partition of *V* with $|{\mathcal {V}}|\ge 2$. Moreover, let ${\mathcal {T}}({\mathcal {V}})$ be the set of trees *T* on *V* that satisfy ${\mathcal {V}} = \{L(T(v)) \mid v\in \mathsf {child}_{T}(\rho _T) \}$. The set of *unsatisfiable relations* (*UR*), denoted by $U(\vec {G},{\mathcal {V}})$, is defined as

3 $$\begin{aligned} U(\vec {G},{\mathcal {V}}) {:}{=}\bigcap _{T\in {\mathcal {T}}({\mathcal {V}})} U(\vec {G},T). \end{aligned}$$

The associated *UR*-cost is $c(\vec {G},{\mathcal {V}}){:}{=}|U(\vec {G},{\mathcal {V}})|$.

The set of (phylogenetic) trees ${\mathcal {T}}({\mathcal {V}})$ is non-empty since $|{\mathcal {V}}|\ge 2$ in Def. 12. Moreover, by construction, $(x,y) \in U(\vec {G},{\mathcal {V}})$ if and only if

$$\begin{aligned} {} &(x,y)\in E \text { and } (x,y)\notin E(\vec {G}(T,\sigma )) \text { for all } T\in {\mathcal {T}}({\mathcal {V}}),\\& \text {or}\\& (x,y)\notin E \text { and } (x,y)\in E(\vec {G}(T,\sigma )) \text { for all } T\in {\mathcal {T}}({\mathcal {V}}). \end{aligned}$$

Intriguingly, the set $U(\vec {G},{\mathcal {V}})$, and thus the *UR*-cost $c(\vec {G},{\mathcal {V}})$, can be computed in polynomial time without any explicit knowledge of the possible trees to determine the set $U(\vec {G},{\mathcal {V}})$. To this end, we define the three sets

$$\begin{aligned} {} & U_1(\vec {G},{\mathcal {V}}) = \bigcup _{V_{i}\in {\mathcal {V}}} \{(x,y) \mid (x,y)\in E,\; x\in V_{i},\; y\in V\setminus V_{i},\\&\quad \sigma (y)\in \sigma (V_{i})\},\\& U_2(\vec {G},{\mathcal {V}}) = \bigcup _{V_{i}\in {\mathcal {V}}} \{(x,y) \mid (x,y)\notin E,\; x\in V_{i},\; y\in V\setminus V_{i},\\&\quad \sigma (y)\notin \sigma (V_{i})\}, \text { and}\\& U_3(\vec {G},{\mathcal {V}}) = \bigcup _{V_{i}\in {\mathcal {V}}} \{(x,y) \mid (x,y)\notin E,\; \text {distinct }x,y\in V_{i},\\&\quad V_{i}[\sigma (y)]=\{y\}\}. \end{aligned}$$

#### Lemma 13

*Let*$(\vec {G}=(V,E),\sigma )$*be a properly vertex-colored digraph and let*${\mathcal {V}}=\{V_1,\dots ,V_k\}$*be a partition ofVwith*$|{\mathcal {V}}|=k\ge 2$. *Then*
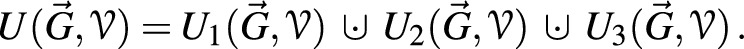


**Fig. 4:**
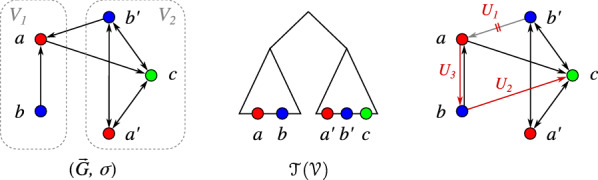
Example for unsatisfiable relations $U(\vec {G},{\mathcal {V}})$ of a vertex-colored digraph $(\vec {G}=(V,E),\sigma )$ w.r.t. a partition ${\mathcal {V}}=\{V_1,V_2\}$ (indicated by the gray boxes). In the middle, the set of trees ${\mathcal {T}}({\mathcal {V}})$ is illustrated, i.e., the triangles represent all possible phylogenetic trees on the respective subset of leaves. On the right, the arc modifications implied by ${\mathcal {V}}$ (i.e., $U(\vec {G},{\mathcal {V}})$) are illustrated where $U_1$, $U_2$, and $U_3$ indicate the type according to Lemma 13

The proof of Lemma 13 relates the possible cases between ${\mathcal {V}}$ and the tree set ${\mathcal {T}}({\mathcal {V}})$ in a straightforward manner. Since it is rather lengthy it is relegated to Appendix. Fig. 4 gives examples for all three types of unsatisfiable relations, i.e., for $U_1(\vec {G},{\mathcal {V}})$, $U_2(\vec {G},{\mathcal {V}})$, and $U_3(\vec {G},{\mathcal {V}})$. In particular, we have $(b', a)\in U_1(\vec {G},{\mathcal {V}})$ since it is an arc in $\vec {G}$ but $V_2$ contains another red vertex $a'$. Moreover, $(b,c)\in U_2(\vec {G},{\mathcal {V}})$ since it is not an arc in $\vec {G}$ but $V_1$ does not contain another green vertex. Finally, we have $(a,b)\in U_3(\vec {G},{\mathcal {V}})$ since it is not an arc in $\vec {G}$ but *b* is the only blue vertex in $V_1$. In the example, the digraph $(\vec {G}{{\,\mathrm{\triangle }\,}}U(\vec {G},{\mathcal {V}}))$ is already a BMG which, however, is not true in general.

#### Corollary 14

*The set* $U(\vec {G},{\mathcal {V}})$ *can be computed in quadratic time.*

#### Proof

We first compute all numbers $n_{i,A}$ of vertices in $V_i$ with a given color *A*. This can be done in *O*(|*V*|) if we do not explicitly store the zero-entries. Now, $\sigma (y)\in \sigma (V_i)$, i.e. $n_{i,\sigma (y)}>0$, can be checked in constant time, and thus, it can also be decided in constant time whether or not a pair (*x*, *y*) is contained in $U_1(\vec {G},{\mathcal {V}})$ or $U_2(\vec {G},{\mathcal {V}})$. Since, given $y\in V_i$, the condition $V_i[\sigma (y)]=\{y\}$ is equivalent to $n_{i,\sigma (y)}=1$, membership in $U_3(\vec {G},{\mathcal {V}})$ can also be decided in constant time. Checking all ordered pairs $x,y\in V$ thus requires a total effort of $O(|V|^2)$. $\square$

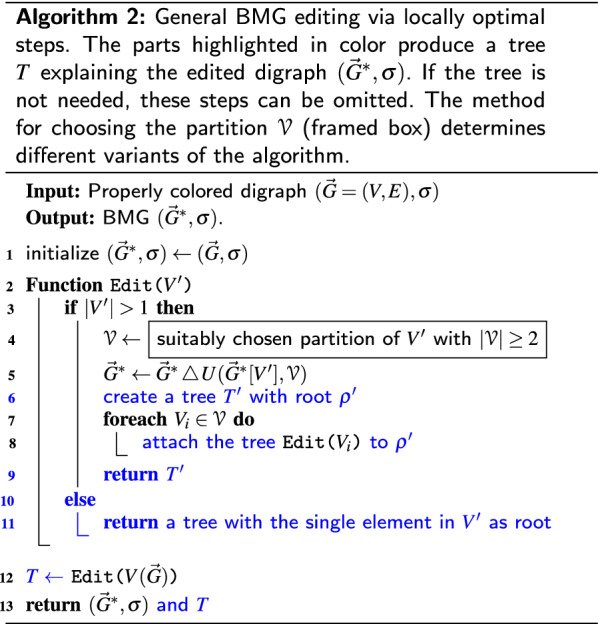



Our discussion so far suggests a recursive top-down approach, made precise in Alg. 2. In each step, one determines a “suitably chosen” partition ${\mathcal {V}}$ and then recurses on the subgraphs of the edited digraph $\vec {G}^* \triangle U(\vec {G}^*[V'],{\mathcal {V}})$. More details on such suitable partitions ${\mathcal {V}}$ will be given in Thm. 23 below. The parts in the algorithm highlighted in color can be omitted. They are useful, however, if one is also interested in a tree $(T,\sigma )$ that explains the editing result $(\vec {G}^*,\sigma )$ and to show that $(\vec {G}^*,\sigma )$ is indeed a BMG (see below). Alg. 2 is designed to accumulate the edit sets in each step, Line 5. In particular, the total edit cost and the scores $c(\vec {G}^*[V'],{\mathcal {V}})$ are closely tied together, which follows from the following result:

#### Lemma 15

*All edit sets* $U(\vec {G}^*[V'],{\mathcal {V}})$ *constructed in Alg. 2 are pairwise disjoint.*

The proof of Lemma 15 and a technical result on which it relies can be found in the Appendix. As an immediate consequence of Lemma 15, we have

#### Corollary 16

*The edit cost of Alg. 2 is the sum of the* *UR* *-costs* $c(\vec {G}^*[V'],{\mathcal {V}})$ *in each recursion step.*

It is important to note that the edits $U(\vec {G}^*[V'],{\mathcal {V}})$ must be applied immediately in each step (cf. Line 5 in Alg. 2). In particular, Lemma 15 and Cor. 16 pertain to the partitioning of the edited digraph $\vec {G}^*$, not to the original digraph $\vec {G}$. We continue by proving the correctness of Alg. 2, i.e., that it returns a valid BMG and a corresponding explaining tree.

#### Theorem 17

*Every pair of edited digraph*$(\vec {G}^*,\sigma )$*and treeTproduced as output by Alg. 2 satisfies*$(\vec {G}^*,\sigma )=\vec {G}(T,\sigma )$. *In particular*, $(\vec {G}^*,\sigma )$*is a* BMG.

#### *Proof*

By construction, the tree *T* is phylogenetic and there is a one-to-one correspondence between the vertices $u\in V(T)$ and the recursion steps, which operate on the sets $V'=L(T(u))$. If $|V'|\ge 2$ (or, equivalently, *u* is an inner vertex of *T*), we furthermore have ${\mathcal {V}}=\{L(T(v)) \mid v\in \mathsf {child}_{T}(u)\}$ for the partition ${\mathcal {V}}$ of $V'$ chosen in that recursion step. In the following, we denote by $(\vec {G}^*,\sigma )$ the digraph during the editing process, and by $(\vec {G},\sigma )$ the input digraph, i.e., as in Alg. 2. For brevity, we write $E^*$ for the arc set of the final edited digraph and $E^T{:}{=}E(\vec {G}(T,\sigma ))$.

Let us assume, for contradiction, that there exists (a) $(x,y)\in E^*\setminus E^T\ne \emptyset$, or (b) $(x,y)\in E^T\setminus E^*\ne \emptyset$. In either case, we set $u{:}{=}{{\,\mathrm{lca}\,}}_T(x,y)$ and consider the recursion step on $V'{:}{=}L(T(u))$ with the corresponding partition ${\mathcal {V}}{:}{=}\{L(T(v)) \mid v\in \mathsf {child}_{T}(u)\}$ chosen for $V'$. Note that $x\ne y$, and thus $u\in V^0(T)$. Moreover, let $v_x$ be the child of *u* such that $x\preceq _{T} v_x$, and $V_x{:}{=}L(T(v_x))\in {\mathcal {V}}$.

Case (a): $(x,y)\in E^*\setminus E^T\ne \emptyset$. Since $(x,y)\notin E^T$ and by the definition of best matches, there must be a vertex $y'\in V_x$ of color $\sigma (y)$ such that ${{\,\mathrm{lca}\,}}_T(x,y')\prec _T{{\,\mathrm{lca}\,}}_{T}(x,y)=u$, and thus $\sigma (y)\in \sigma (V_x)$. Moreover, we have $V_x\in {\mathcal {V}}$, $x\in V_x$ and $y\in V'\setminus V_x$. Two subcases need to be considered, depending on whether or not (*x*, *y*) is an arc in $\vec {G}^*$ at the beginning of the recursion step. In the first case, the arguments above imply that $(x,y)\in U_1(\vec {G}^*[V'], {\mathcal {V}})$, and thus, $(x,y)\in U(\vec {G}^*[V'], {\mathcal {V}})$ by Lemma 13. Hence, we delete the arc (*x*, *y*) in this step. In the second case, it is an easy task to verify that none of the definitions of $U_1(\vec {G}^*[V'], {\mathcal {V}})$, $U_2(\vec {G}^*[V'], {\mathcal {V}})$, and $U_3(\vec {G}^*[V'], {\mathcal {V}})$ matches for (*x*, *y*). Since this step is clearly the last one in the recursion hierarchy that can affect the (non-)arc (*x*, *y*), it follows for both subcases that $(x,y)\notin E^*$; a contradiction.

Case (b): $(x,y)\in E^T\setminus E^*\ne \emptyset$. Since $(x,y)\in E^T$ and by the definition of best matches, there cannot be a vertex $y'\in V_x$ of color $\sigma (y)$ such that ${{\,\mathrm{lca}\,}}_T(x,y')\prec _T{{\,\mathrm{lca}\,}}_{T}(x,y)=u$, and thus $\sigma (y)\notin \sigma (V_x)$. Moreover, we have $V_x\in {\mathcal {V}}$, $x\in V_x$ and $y\in V'\setminus V_x$. Again, two subcases need to be distinguished depending on whether or not (*x*, *y*) is an arc in $\vec {G}^*$ at the beginning of the recursion step. In the first case, the arguments above make it easy to verify that none of the definitions of $U_1(\vec {G}^*[V'], {\mathcal {V}})$, $U_2(\vec {G}^*[V'], {\mathcal {V}})$, and $U_3(\vec {G}^*[V'], {\mathcal {V}})$ matches for (*x*, *y*). In the second case, we obtain $(x,y)\in U_2(\vec {G}^*[V'], {\mathcal {V}})$, and thus, $(x,y)\in U(\vec {G}^*[V'], {\mathcal {V}})$ by Lemma 13. Hence, we insert the arc (*x*, *y*) in this step. As before, the (non-)arc (*x*, *y*) remains unaffected in any deeper recursion step. Therefore, we have $(x,y)\in E^*$ in both subcases; a contradiction.

Finally, $(\vec {G}^*,\sigma )=\vec {G}(T,\sigma )$ immediately implies that $(\vec {G}^*,\sigma )$ is a BMG. $\square$

**Fig. 5:**
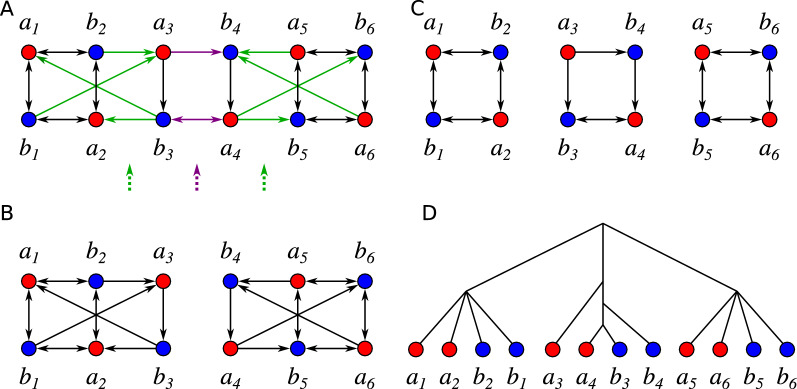
**A** Example for a colored digraph $(\vec {G},\sigma )$ in which the “locally” optimal (first) split does not result in a global optimal BMG editing. The minimal *UR*-cost equals 3 and is attained only for the partition ${\mathcal {V}}=\{ \{a_1,a_2,a_3,b_1,b_2,b_3\}, \{a_4,a_5,a_6,b_4,b_5,b_6\}\}$, which was verified by full enumeration of all partitions and Lemma 13. For this partition, $U(\vec {G},{\mathcal {V}})$ comprises the three purple arcs. **B** The two (isomorphic) induced subgraphs obtained by applying the locally optimal partition ${\mathcal {V}}$. Each of them has a (global) optimal BMG editing cost of 4. Therefore, the overall symmetric difference of an edited digraph (using the initial split ${\mathcal {V}}$ as specified) comprises at least $c(\vec {G},{\mathcal {V}})+2\cdot 4=11$ arcs. **C** An optimal editing removes the 8 green arcs and results in a digraph that is explained by the tree in **D**. The optimality of this solution was verified using an implementation of the ILP formulation for BMG editing given in [8]

Cor. 16 suggests a greedy-like “local” approach. In each step, the partition ${\mathcal {V}}$ is chosen to minimize the score $c(\vec {G}, {\mathcal {V}})$ in Line 4. The example in Fig. 5 shows, however, that the greedy-like choice of ${\mathcal {V}}$ does not necessarily yield a globally optimal edit set.

In order to identify arcs that must be contained in every edit set, we first clarify the relationship between the partitions ${\mathfrak {P}}_{\ge 2}$ on *V* and the partitions defined by the phylogenetic trees on *V*.

#### Lemma 18

*LetVbe a set with*$|V|\ge 2$. *Let*${\mathfrak {P}}_{\ge 2}$*be the set of all partitions*${\mathcal {V}}$*ofVwith*$|{\mathcal {V}}|\ge 2$. *Then the set*${\mathcal {T}}_V$*of all phylogenetic trees with leaf setVsatisfies*${\mathcal {T}}_V= \bigcup _{{\mathcal {V}}\in {\mathfrak {P}}_{\ge 2}} {\mathcal {T}}({\mathcal {V}})$.

#### Proof

For every ${\mathcal {V}}\in {\mathfrak {P}}_{\ge 2}$, ${\mathcal {T}}({\mathcal {V}})$ is a set of phylogenetic trees on *V*. Hence, we conclude $\bigcup _{{\mathcal {V}}\in {\mathfrak {P}}_{\ge 2}} {\mathcal {T}}({\mathcal {V}}) \subseteq {\mathcal {T}}_V$. Conversely, assume that $T\in {\mathcal {T}}_V$. Since *T* (with root $\rho _T$) is a phylogenetic tree and has at least two leaves, we have $|\mathsf {child}_{T}(\rho _T)|\ge 2$. Together with $L(T(\rho _T))=L(T)=V$, this implies ${\mathcal {V}}^*{:}{=}\{L(T(v)) \mid v\in \mathsf {child}_{T}(\rho _T)\} \in {\mathfrak {P}}_{\ge 2}$. In particular, *T* satisfies $T\in {\mathcal {T}}({\mathcal {V}}^*)$ for some ${\mathcal {V}}^* \in {\mathfrak {P}}_{\ge 2}$, and is therefore contained in $\bigcup _{{\mathcal {V}}\in {\mathfrak {P}}_{\ge 2}} {\mathcal {T}}({\mathcal {V}})$. $\square$

Using Lemma 18 and given that $|V|\ge 2$, we can express the set of relations that are unsatisfiable for every partition as follows

4 $$\begin{aligned} \begin{aligned} \bigcap _{{\mathcal {V}}\in {\mathfrak {P}}_{\ge 2}} U(\vec {G},{\mathcal {V}})&= \bigcap _{{\mathcal {V}}\in {\mathfrak {P}}_{\ge 2}} \left( \bigcap _{T\in {\mathcal {T}}({\mathcal {V}})} U(\vec {G},T) \right) \\[5pt]&= \bigcap _{T\in \bigcup _{{\mathcal {V}}\in {\mathfrak {P}}_{\ge 2}} {\mathcal {T}}({\mathcal {V}})} U(\vec {G},T)\\&= \bigcap _{T\in {\mathcal {T}}_V} U(\vec {G},T) = U^*(\vec {G})\;, \end{aligned} \end{aligned}$$

i.e., it coincides with the set of relations that are unsatisfiable for every phylogenetic tree, and thus part of every edit set. Note that $U^*(\vec {G})$ is trivially empty if $|V|<2$. We next show that $U^*(\vec {G})$ can be computed without considering the partitions of *V* explicitly.

#### Theorem 19

*Let* $(\vec {G}=(V,E),\sigma )$ *be a properly vertex-colored digraph with* $|V|\ge 2$ *then*

5 $$\begin{aligned} U^*(\vec {G}) = \left\{ (x,y) \mid (x,y)\notin E,\; x\ne y,\; V[\sigma (y)]=\{y\} \right\} . \end{aligned}$$

#### *Proof*

First note that $|V|\ge 2$ ensures that ${\mathfrak {P}}_{\ge 2}\ne \emptyset$. Moreover, since $|{\mathcal {V}}|\ge 2$ for any ${\mathcal {V}}\in {\mathfrak {P}}_{\ge 2}$, the sets ${\mathcal {T}}({\mathcal {V}})$ are all non-empty as well. With the abbreviation ${\hat{U}}(\vec {G})$ for the right-hand side of Eq. (5), we show that ${\hat{U}}(\vec {G})= \bigcap _{{\mathcal {V}}\in {\mathfrak {P}}_{\ge 2}} U(\vec {G},{\mathcal {V}})$ which by Eq. (4) equals $U^*(\vec {G})$.

Suppose that $(x,y)\in {\hat{U}}(\vec {G})$. Then $x\ne y$ and $V[\sigma (y)]=\{y\}$ imply that $\sigma (x)\ne \sigma (y)$. This together with the facts that (i) *y* is the only vertex of its color in *V*, and (ii) $L(T)=V$ for each $T\in {\mathcal {T}}({\mathcal {V}})$ and any ${\mathcal {V}}\in {\mathfrak {P}}_{\ge 2}$ implies that *y* is a best match of *x* in every such tree *T*, i.e. $(x,y)\in E(\vec {G}(T,\sigma ))$. Since in addition $(x,y)\notin E$ by assumption, we conclude that $(x,y)\in U^{*}(\vec {G})$.

Now suppose that $(x,y)\in U^{*}(\vec {G})$. Observe that $\sigma (x)\ne \sigma (y)$ (and thus $x\ne y$) as a consequence of Def. 12 and the fact that $(\vec {G},\sigma )$ and all BMGs are properly colored. If $V=\{x,y\}$ and thus $\{\{x\},\{y\}\}$ is the only partition in ${\mathfrak {P}}_{\ge 2}$, the corresponding unique tree *T* consists of *x* and *y* connected to the root. In this case, we clearly have $(x,y)\in E(\vec {G}(T,\sigma ))$ since $\sigma (x)\ne \sigma (y)$. On the other hand, if $\{x,y\}\subsetneq V$, then we can find a partition ${\mathcal {V}}\in {\mathfrak {P}}_{\ge 2}$ such that $V_{i}=\{x,y\}$ for some $V_{i}\in {\mathcal {V}}$. In this case, every tree $T\in {\mathcal {T}}({\mathcal {V}})$ has a vertex $v_{i}\in \mathsf {child}_{T}(\rho _T)$ with the leaves *x* and *y* as its single two children. Clearly, $(x,y)\in E(\vec {G}(T,\sigma ))$ holds for any such tree. In summary, there always exists a partition ${\mathcal {V}}\in {\mathfrak {P}}_{\ge 2}$ such that $(x,y)\in E(\vec {G}(T,\sigma ))$ for some tree $T\in {\mathcal {T}}({\mathcal {V}})$. Therefore, by $(x,y)\in \bigcap _{{\mathcal {V}}\in {\mathfrak {P}}_{\ge 2}} U(\vec {G},{\mathcal {V}})$ and Def. 12, we conclude that $(x,y)\notin E$. In order to obtain $(x,y)\in {\hat{U}}(\vec {G})$, it remains to show that $V[\sigma (y)]=\{y\}$. Since $(x,y)\notin E$ and $(x,y)\in \bigcap _{{\mathcal {V}}\in {\mathfrak {P}}_{\ge 2}} U(\vec {G},{\mathcal {V}})$, it must hold that $(x,y)\in E(\vec {G}(T,\sigma ))$ for all $T\in {\mathcal {T}}({\mathcal {V}})$ and all ${\mathcal {V}}\in {\mathfrak {P}}_{\ge 2}$. Now assume, for contradiction, that there is a vertex $y'\ne y$ of color $\sigma (y')=\sigma (y)$. Since $\sigma (x)\ne \sigma (y)$, the vertices $x,y,y'$ must be pairwise distinct. Hence, we can find a partition ${\mathcal {V}}\in {\mathfrak {P}}_{\ge 2}$ such that $V_{i}=\{x,y'\}$ for some $V_{i}\in {\mathcal {V}}$. In this case, every tree $T\in {\mathcal {T}}({\mathcal {V}})$ has a vertex $v_{i}\in \mathsf {child}_{T}(\rho _T)$ with only the leaves *x* and $y'$ as its children. Clearly, ${{\,\mathrm{lca}\,}}_T(x,y')=v_{i}\prec _{T}\rho _{T}={{\,\mathrm{lca}\,}}_{T}(x,y)$, and thus $(x,y)\notin E(\vec {G}(T,\sigma ))$; a contradiction. Therefore, we conclude that *y* is the only vertex of its color in *V*, and hence, $(x,y)\in {\hat{U}}(\vec {G})$. In summary, therefore, we have $U^{*}(\vec {G})={\hat{U}}(\vec {G})$. $\square$

As a consequence of Thm. 19 and by similar arguments as in the proof of Cor. 14, we observe

#### Corollary 20

*The set* $U^*(\vec {G})$ *can be computed in quadratic time.*

By Thm. 19, $U^*(\vec {G})$ contains only non-arcs, more precisely, missing arcs pointing towards a vertex that is the only one of its color and thus, by definition, a best match of every other vertex irrespective of the details of the gene tree. By definition, furthermore, $U^*(\vec {G})$ is a subset of every edit set for $(\vec {G},\sigma )$. We therefore have the lower bound

6 $$\begin{aligned} |U^*(\vec {G})| \le c(\vec {G},{\mathcal {V}}) \end{aligned}$$

for every $${\mathcal {V}}\in {\mathfrak {P}}_{\ge 2}$$.

The following result shows that if $(\vec {G},\sigma )$ is a BMG, then a suitable partition ${\mathcal {V}}$ can be chosen such that $c(\vec {G},{\mathcal {V}})=|U^*(\vec {G})|=0$.

#### Lemma 21

*Let*$(\vec {G}=(V,E),\sigma )$*be a BMG with*$|V|\ge 2$*and*${\mathcal {V}}$*be the connected components of the Aho graph*$[{\mathcal {R}}(\vec {G},\sigma ), V]$. *Then the partition*${\mathcal {V}}$*ofVsatisfies*$|{\mathcal {V}}|\ge 2$*and*$c(\vec {G},{\mathcal {V}})=0$.

#### *Proof*

Since $(\vec {G},\sigma )$ is a BMG, we can apply Prop. 10 to conclude that ${\mathcal {R}}{:}{=}{\mathcal {R}}(\vec {G},\sigma )$ is consistent and that $(T,\sigma ){:}{=}({{\,\mathrm{Aho}\,}}({\mathcal {R}}, V),\sigma )$ explains $(\vec {G},\sigma )$, i.e., $\vec {G}(T,\sigma )=(\vec {G},\sigma )$. Hence, $U(\vec {G},T)=\emptyset$. From $|V|\ge 2$ and consistency of ${\mathcal {R}}$, it follows that $[{\mathcal {R}}, V]$ has at least two connected components [10], and thus, by construction, $|{\mathcal {V}}|\ge 2$. Moreover, we clearly have $T\in {\mathcal {T}}({\mathcal {V}})$ by the construction of *T* via BUILD. Together with $U(\vec {G},T)=\emptyset$, the latter implies $U(\vec {G},{\mathcal {V}})=\emptyset$, and thus $c(\vec {G},{\mathcal {V}})=0$. $\square$

#### Lemma 22

*Let*$(\vec {G}=(V,E),\sigma )$*be a BMG, and*${\mathcal {V}}$*a partition ofVsuch that*$c(\vec {G},{\mathcal {V}})=0$. *Then the induced subgraph*$(\vec {G}[V'],\sigma _{|V'})$*is a BMG for every*$V'\in {\mathcal {V}}$.

#### *Proof*

Set ${\mathcal {R}}{:}{=}{\mathcal {R}}(\vec {G},\sigma )$ and ${\mathcal {F}}{:}{=}{\mathcal {F}}(\vec {G},\sigma )$ for the sets of informative and forbidden triples of $(\vec {G},\sigma )$, respectively. Since $(\vec {G},\sigma )$ is a BMG, we can apply Prop. 10 to conclude that $({\mathcal {R}},{\mathcal {F}})$ is consistent. Now we choose an arbitrary set $V'\in {\mathcal {V}}$ and set $(\vec {G}',\sigma '){:}{=}(\vec {G}[V'],\sigma _{|V'})$. By Obs. 8, we obtain ${\mathcal {R}}(\vec {G}',\sigma ')={\mathcal {R}}_{|V'}$ and ${\mathcal {F}}(\vec {G}',\sigma ')={\mathcal {F}}_{|V'}$. This together with the fact that ${\mathcal {R}}_{|V'}\subseteq {\mathcal {R}}$ and ${\mathcal {F}}_{|V'}\subseteq {\mathcal {F}}$ and Obs. 9 implies that $({\mathcal {R}}_{|V'},{\mathcal {F}}_{|V'})=({\mathcal {R}}(\vec {G}',\sigma '), {\mathcal {F}}(\vec {G}',\sigma '))$ is consistent.

By Prop. 10, it remains to show that $(\vec {G}',\sigma ')$ is sf-colored to prove that it is a BMG. To this end, assume for contradiction that there is a vertex $x\in V'$ and a color $s\in \sigma (V')$ such that *x* has no out-neighbor of color $s\ne \sigma (x)$ in $V'$. However, since the color *s* is contained in $\sigma (V)$ and $(\vec {G},\sigma )$ is a BMG, and thus sf-colored, we conclude that there must be a vertex $y\in V\setminus V'$ of color *s* such that $(x,y)\in E$. In summary, we obtain $(x,y)\in E$, $x\in V'$, $y\in V\setminus V'$ and $\sigma (y)=s\in \sigma (V')$. Thus, we have $(x,y)\in U_1(\vec {G},{\mathcal {V}})$. Hence, Lemma 13 implies that $(x,y)\in U(\vec {G},{\mathcal {V}})$ and, hence, $c(\vec {G},{\mathcal {V}})>0$; a contradiction. Therefore, $(\vec {G}',\sigma ')$ must be sf-colored, which concludes the proof. $\square$

Lemma 21 and 22 allow us to choose the partition ${\mathcal {V}}$ in each step of Alg. 2 in such a way that Alg. 2 is consistent, i.e., BMGs remain unchanged.

#### Theorem 23

*Alg. 2 is consistent if, in each step on*$V'$*with*$|V'|\ge 2$, *the partition*${\mathcal {V}}$*in Line* 4 *is chosen according to* *one of the following rules:*

1. ${\mathcal {V}}$ has minimal *UR*-cost among all possible partitions ${\mathcal {V}}'$ of $V'$ with $|{\mathcal {V}}'|\ge 2$.
2. If the Aho graph $[{\mathcal {R}}(\vec {G}^*[V'],\sigma _{|V'}),V']$ is disconnected with the set of connected components ${\mathcal {V}}_{{{\,\mathrm{Aho}\,}}}$, and moreover $c(\vec {G}^*[V'],{\mathcal {V}}_{{{\,\mathrm{Aho}\,}}})=0$, then ${\mathcal {V}}={\mathcal {V}}_{{{\,\mathrm{Aho}\,}}}$.

#### *Proof*

We have to show that the final edited digraph $(\vec {G}^*,\sigma )$ returned in Line 13 equals the input digraph $(\vec {G}=(V,E),\sigma )$ whenever $(\vec {G},\sigma )$ already is a BMG, i.e., nothing is edited. Thus suppose that $(\vec {G},\sigma )$ is a BMG and first consider the top-level recursion step on *V* (where initially $\vec {G}^*=\vec {G}$ still holds at Line 1). If $|V|=1$, neither $(\vec {G},\sigma )$ nor $(\vec {G}^*,\sigma )$ contain any arcs, and thus, the edit cost is trivially zero. Now suppose $|V|\ge 2$. Since $(\vec {G},\sigma )$ is a BMG, Lemma 21 guarantees the existence of a partition ${\mathcal {V}}$ satisfying $c(\vec {G},{\mathcal {V}})=0$, in particular, the connected components ${\mathcal {V}}_{{{\,\mathrm{Aho}\,}}}$ of the Aho graph $[{\mathcal {R}}(\vec {G},\sigma ), V]$ form such a partition. Hence, for both rules (1) and (2), we choose a partition ${\mathcal {V}}$ with (minimal) *UR*-cost $c(\vec {G},{\mathcal {V}})=0$. Now, Lemma 22 implies that the induced subgraph $(\vec {G}[V'],\sigma _{|V'})$ is a BMG for every $V'\in {\mathcal {V}}$. Since we recurse on these subgraphs, we can repeat the arguments above along the recursion hierarchy to conclude that the *UR*-cost $c(\vec {G}^*[V'],{\mathcal {V}}')$ vanishes in every recursion step. By Cor. 16, the total edit cost of Alg. 2 is the sum of the *UR*-costs $c(\vec {G}^*[V'],{\mathcal {V}}')$ in each recursion step, and thus, also zero. Therefore, we conclude that we still have $(\vec {G}^*,\sigma )=(\vec {G},\sigma )$ in Line 13. $\square$

By Thm. 23, Alg. 2 is consistent whenever the choice of ${\mathcal {V}}$ minimizes the *UR*-cost of ${\mathcal {V}}$ in each step. We shall see below that minimizing $c(\vec {G},{\mathcal {V}})$ is a difficult optimization problem in general. Therefore, a good heuristic will be required for this step. This, however, may not guarantee consistency of Alg. 2 in general. The second rule in Thm. 23 provides a remedy: the Aho graph $[{\mathcal {R}}(\vec {G}^*[V'],\sigma _{|V'}), V']$ can be computed efficiently. Whenever $[{\mathcal {R}}(\vec {G}^*[V'],\sigma _{|V'}), V']$ is not connected, the partition ${\mathcal {V}}_{{{\,\mathrm{Aho}\,}}}$ defined by the connected components $[{\mathcal {R}}(\vec {G}^*[V'],\sigma _{|V'}), V']$ is chosen provided it has *UR*-cost zero. This procedure is effectively a generalization of the algorithm BUILD using as input the set of informative triples ${\mathcal {R}}(\vec {G},\sigma )$ of a properly vertex-colored digraph $(\vec {G},\sigma )$. If $(\vec {G},\sigma )$ is already a BMG, then the recursion in Alg. 2 is exactly the same as in BUILD: it recurses on the connected components of the Aho graph (cf. Prop. 10). We can summarize this discussion as

#### Corollary 24

$(\vec {G},\sigma )$*is a BMG if and only if, in every step of the*BUILD*algorithm operating on*${\mathcal {R}}(\vec {G},\sigma )_{|V'}$*and*$V'$, *either*$|V'|=1$, *or*$c(\vec {G}^*[V'], {\mathcal {V}}_{{{\,\mathrm{Aho}\,}}})=0$*for the connected componentpartition*${\mathcal {V}}_{{{\,\mathrm{Aho}\,}}}$*of the disconnected Aho graph*$[{\mathcal {R}}(\vec {G}^*[V'],\sigma _{|V'}), V']$.

For recursion steps in which the Aho graph is connected, and possibly also in steps with non-zero *UR*-cost, another (heuristic) rule has to be employed. As a by-product, we obtain an approach for the case that ${\mathcal {R}}(\vec {G},\sigma )$ is consistent: Following BUILD yields the approximation $\vec {G}({{\,\mathrm{Aho}\,}}({\mathcal {R}}(\vec {G},\sigma ),V(\vec {G})),\sigma )$ as a natural choice.

### Binary-explainable BMGs

Phylogenetic trees are often binary. Multifurcations are in many cases – but not always – the consequence of insufficient data [14, 20, 21]. It is therefore of practical interest to consider BMGs that can be explained by a binary tree:

#### Definition 25

A properly colored digraph $(\vec {G},\sigma )$ is a *binary-explainable best match graph* (*beBMG*) if there is a binary tree *T* such that $\vec {G}(T,\sigma )=(\vec {G},\sigma )$.

Correspondingly, it is of interest to edit a properly colored digraph to a beBMG, which translates to the following decision problem:

#### Problem 2

($\ell$-BMG Editing restricted to Binary-Explainable Graphs (EBEG))

**Table:** 

*Input:*	A properly $\ell$-colored digraph
	$(\vec {G}=(V,E),\sigma )$ and an integer *k*.
*Question:*	Is there a subset
	$F\subseteq V\times V \setminus \{(v,v)\mid v\in V\}$ such that
	$|F|\le k$ and $(\vec {G}{{\,\mathrm{\triangle }\,}}F,\sigma )$ is a binary-
	explainable $\ell$-BMG?

We call the corresponding completion and deletion problem $\ell$-BMG CBEG and $\ell$-BMG DBEG, respectively. As their more general counterparts, all three variants are NP-complete as well, cf. [8, Cor. 6.2] and [14, Thm. 5].

Since the recursive partitioning in Alg. 2 defines a tree that explains the edited BMG, see Thm. 17, it is reasonable to restrict the optimization of ${\mathcal {V}}$ in Line 17 to bipartitions. The problem still remains hard, however, since the corresponding decision problem (problem BPURC) is NP-complete as shown in Thm. 30 below. Similar to BMGs in general, beBMGs have a characterization in terms of informative triples:

#### Proposition 26

[14, Thm. 3.5] *A properly vertex-colored digraph* $(\vec {G},\sigma )$ *with vertex set* *V* *is binary-explainable if and only* *if (i)* $(\vec {G},\sigma )$ *is sf-colored, and (ii) the triple set* $\mathop {{\mathcal {R}}^{\text {B}}}(\vec {G},\sigma )$ *is consistent. In this case, the BMG* $(\vec {G},\sigma )$ *is explained by every refinement of the* *binary refinable tree* $({{\,\mathrm{Aho}\,}}(\mathop {{\mathcal {R}}^{\text {B}}}(\vec {G},\sigma ), V), \sigma )$.

Using Prop. 26, we can apply analogous arguments as in the proof of Lemma 21 for $\mathop {{\mathcal {R}}^{\text {B}}}(\vec {G},\sigma )$ instead of ${\mathcal {R}}(\vec {G},\sigma )$ to obtain

#### Corollary 27

*Let*$(\vec {G}=(V,E),\sigma )$*be a beBMG with*$|V|\ge 2$*and*${\mathcal {V}}$*be the connected components of the Aho graph*$[\mathop {{\mathcal {R}}^{\text {B}}}(\vec {G},\sigma ), V]$. *Then the partition*${\mathcal {V}}$*ofVsatisfies*$|{\mathcal {V}}|\ge 2$*and*$c(\vec {G},{\mathcal {V}})=0$.

Since a beBMG $(\vec {G},\sigma )$ is explained by every refinement of the Aho tree constructed from $\mathop {{\mathcal {R}}^{\text {B}}}(\vec {G},\sigma )$ (cf. Prop. 26), we can obtain a slightly more general result.

#### Lemma 28

*Let*$(\vec {G}=(V,E),\sigma )$*be a beBMG with*$|V|\ge 2$*and*${\mathcal {V}}$*be the connected components of the Aho graph*$[\mathop {{\mathcal {R}}^{\text {B}}}(\vec {G},\sigma ),V]$. *Then, every coarse-graining*${\mathcal {V}}'$*of*${\mathcal {V}}$*with*$|{\mathcal {V}}'|\ge 2$*satisfies*$c(\vec {G},{\mathcal {V}}')=0$.

#### *Proof*

First note that $\mathop {{\mathcal {R}}^{\text {B}}}(\vec {G},\sigma )$ is consistent by Prop. 26 since $(\vec {G},\sigma )$ is a beBMG. Therefore, $|V|\ge 2$ implies $|{\mathcal {V}}|\ge 2$ [10]. For the trivial coarse-graining ${\mathcal {V}}'={\mathcal {V}}$, Cor. 27 already implies the statement. Now assume ${\mathcal {V}}'\ne {\mathcal {V}}$. Observe that the tree $(T,\sigma ) {:}{=}({{\,\mathrm{Aho}\,}}(\mathop {{\mathcal {R}}^{\text {B}}}(\vec {G},\sigma ), V), \sigma )$ exists and explains $(\vec {G},\sigma )$ by Prop. 26. Moreover, there is, by construction, a one-to-one correspondence between the children $v_i$ of its root $\rho$ and the elements in $V_i\in {\mathcal {V}}$ given by $L(T(v_i))=V_i$. We construct a refinement (tree) $T'$ of *T* as follows: Whenever we have multiple sets $V_i\in {\mathcal {V}}$ that are subsets of the same set $V_j\in {\mathcal {V}}'$, we remove the edges $\rho v_i$ to the corresponding vertices $v_i\in \mathsf {child}_{T}(\rho )$ in *T*, and collectively connect these $v_i$ to a newly created vertex $w_j$. These vertices $w_j$ are then reattached to the root $\rho$. Since $|{\mathcal {V}}'|\ge 2$ by assumption, the so-constructed tree $T'$ is still phylogenetic. Moreover, it satisfies ${\mathcal {V}}'=\{L(T'(v)) \mid v\in \mathsf {child}_{T'}(\rho )\}$, and thus, $T'\in {\mathcal {T}}({\mathcal {V}}')$. It is a refinement of *T* since contraction of the edges $\rho w_j$ again yields *T*. Hence, we can apply Prop. 26 to conclude that $(T',\sigma )$ also explains $(\vec {G},\sigma )$. It follows immediately that $U(\vec {G},T')=\emptyset$. The latter together with $T'\in {\mathcal {T}}({\mathcal {V}}')$ implies $U(\vec {G},{\mathcal {V}}')=\emptyset$, and thus $c(\vec {G},{\mathcal {V}}')=0$. $\square$

We are now in the position to formulate an analogue of Thm. 23 for variants of Alg. 2 that aim to edit a properly-colored digraph $(\vec {G},\sigma )$ to a beBMG.

#### Theorem 29

*Alg. 2 is consistent for beBMGs*$(\vec {G},\sigma )$*if, in each step on*$V'$*with*$|V'|\ge 2$, *a bipartition*${\mathcal {V}}$*in Line* 4 *is chosen according to one of the following rules:*

1. ${\mathcal {V}}$ has minimal *UR*-cost among all possible bipartitions ${\mathcal {V}}'$ of $V'$.
2. If the Aho graph $[\mathop {{\mathcal {R}}^{\text {B}}}(\vec {G}^*[V'],\sigma _{|V'}),V']$ is disconnected with the set of connected components ${\mathcal {V}}_{{{\,\mathrm{Aho}\,}}}$, and moreover $c(\vec {G}^*[V'],{\mathcal {V}}_{{{\,\mathrm{Aho}\,}}})=0$, then ${\mathcal {V}}$ is a coarse-graining of ${\mathcal {V}}_{{{\,\mathrm{Aho}\,}}}$.

#### *Proof*

We have to show that the final edited digraph $(\vec {G}^*,\sigma )$ returned in Line 13 equals the input digraph $(\vec {G}=(V,E),\sigma )$ whenever $(\vec {G},\sigma )$ already is a beBMG, i.e., nothing is edited. Thus suppose that $(\vec {G},\sigma )$ is a beBMG and first consider the top-level recursion step on *V* (where initially $\vec {G}^*=\vec {G}$ still holds at Line 1). If $|V|=1$, neither $(\vec {G},\sigma )$ nor $(\vec {G}^*,\sigma )$ contain any arcs, and thus, the edit cost is trivially zero. Now suppose $|V|\ge 2$. Since $(\vec {G},\sigma )$ is a beBMG, $\mathop {{\mathcal {R}}^{\text {B}}}{:}{=}\mathop {{\mathcal {R}}^{\text {B}}}(\vec {G},\sigma )$ is consistent, and thus, the set of connected components ${\mathcal {V}}_{{{\,\mathrm{Aho}\,}}}$ of the Aho graph $[\mathop {{\mathcal {R}}^{\text {B}}}, V]$ has a cardinality of at least two. If $|{\mathcal {V}}_{{{\,\mathrm{Aho}\,}}}|=2$, ${\mathcal {V}}{:}{=}{\mathcal {V}}_{{{\,\mathrm{Aho}\,}}}$ is a bipartition satisfying $c(\vec {G},{\mathcal {V}})=0$ by Cor. 27. If $|{\mathcal {V}}_{{{\,\mathrm{Aho}\,}}}|>2$, we can find an arbitrary bipartition ${\mathcal {V}}$ that is a coarse-graining of ${\mathcal {V}}_{{{\,\mathrm{Aho}\,}}}$. By Lemma 28, ${\mathcal {V}}$ also satisfies $c(\vec {G},{\mathcal {V}})=0$ in this case. Hence, for both rules (1) and (2), we choose a bipartition ${\mathcal {V}}$ with (minimal) *UR*-cost $c(\vec {G},{\mathcal {V}})=0$. Now, Lemma 22 implies that the induced subgraph $(\vec {G}[V'],\sigma _{|V'})$ is a BMG for every $V'\in {\mathcal {V}}$. To see that $(\vec {G}[V'],\sigma _{|V'})$ is also binary-explainable, first note that $\mathop {{\mathcal {R}}^{\text {B}}}(\vec {G}[V'],\sigma _{|V'})=\mathop {{\mathcal {R}}^{\text {B}}}_{|V'}$ by Obs. 8. This together with the fact that $\mathop {{\mathcal {R}}^{\text {B}}}_{|V'}\subseteq \mathop {{\mathcal {R}}^{\text {B}}}$ and Obs. 9 implies that $\mathop {{\mathcal {R}}^{\text {B}}}(\vec {G}[V'],\sigma _{|V'})$ is consistent. Moreover, Prop. 10 and $(\vec {G}[V'],\sigma _{|V'})$ being a BMG together imply that $(\vec {G}[V'],\sigma _{|V'})$ is sf-colored. Hence, we can apply Prop. 26 to conclude that $(\vec {G}[V'],\sigma _{|V'})$ is a beBMG.

Since we recurse on the subgraphs $(\vec {G}[V'],\sigma _{|V'})$, which are again beBMGs, we can repeat the arguments above along the recursion hierarchy to conclude that the *UR*-cost $c(\vec {G}^*[V'],{\mathcal {V}}')$ vanishes in every recursion step. By Cor. 16, the total edit cost of Alg. 2 is the sum of the *UR*-costs $c(\vec {G}^*[V'],{\mathcal {V}}')$ in each recursion step, and thus, also zero. Therefore, we conclude that we still have $(\vec {G}^*,\sigma )=(\vec {G},\sigma )$ in Line 13. $\square$

### Minimizing the *UR*-cost $c(\vec {G},{\mathcal {V}})$

The problem of minimizing $c(\vec {G},{\mathcal {V}})$ for a given properly colored digraph $(\vec {G},\sigma )$ corresponds to the following decision problem.

#### Problem 3

((Bi)Partition with *UR*-Cost ((B)PURC))

**Table:** 

*Input:*	A properly $\ell$-colored digraph
	$(\vec {G}=(V,E),\sigma )$ and an integer $k\ge 0$.
*Question:*	Is there a (bi)partition ${\mathcal {V}}$ of *V*
	such that $c(\vec {G},{\mathcal {V}})\le k$?

In the Appendix, we show that (B)PURC is NP-hard by reduction from Set Splitting, one of Garey and Johnson’s [22] classical NP-complete problems.

#### Theorem 30

BPURC *is NP-complete.*

Thm. 23 suggests to consider heuristics for (B)PURC that make use of the Aho graph in the following manner:

1. Construct the Aho graph $H{:}{=}[{\mathcal {R}}(\vec {G},\sigma ),V]$ based on the set of informative triples ${\mathcal {R}}(\vec {G},\sigma )$.
2. If *H* has more than one connected component, we use the set of connected components as the partition ${\mathcal {V}}$.
3. If *H* is connected, a heuristic that operates on the Aho graph *H* is used to find a partition ${\mathcal {V}}$ with small *UR*-cost $c(\vec {G},{\mathcal {V}})$.

Plugging any algorithm of this type into Line 4 of Alg. 2 reduces the algorithm to BUILD if a BMG is used as input and thus guarantees consistency (cf. Prop. 10). We note, however, that the connected components of a disconnected Aho graph are not guaranteed to correspond to an optimal solution for (B)PURC in the general case.

## Methods

### Construction of test instances

We test the heuristics described below on ensembles of perturbed BMGs that are constructed as follows: We first generate leaf-colored trees $(T,\sigma )$ with a predefined number of vertices *N* and colors $\ell$ and then compute their BMGs $\vec {G}(T,\sigma )$. To construct the tree *T*, we start from a single vertex. We then repeatedly choose one of the existing vertices *v* randomly, and, depending on whether *v* is currently an inner vertex or a leaf, attach either a single or two new leaves to it, respectively. Hence, the number of leaves increases by exactly one and the tree remains phylogenetic in each step. We stop when the desired number *N* of leaves is reached. In the next step, colors are assigned randomly to the leaves under the constraint that each of the $\ell$ colors appears at least once. We note that trees created in this manner are usually not least resolved, and their BMGs are in general not binary-explainable. Finally, we disturb these BMGs by inserting and deleting arcs according to a specified insertion and deletion probability, respectively. Since arcs between vertices of the same color trivially cannot correspond to best matches, we do not insert arcs between such vertices, i.e., the input digraphs for the editing are all properly vertex-colored digraphs.

For the purpose of benchmarking the heuristics for the (B)PURC problem, we only retain perturbed BMGs $(\vec {G},\sigma )$ with a connected Aho graph $H{:}{=}[{\mathcal {R}}(\vec {G},\sigma ), V(\vec {G})]$ because the heuristics are not applied to instances with a disconnected Aho graph *H*. Depending on the insertion and deletion probabilities, we retained 93% to 100% of the initial sample, except in the case where arcs were only inserted to obtain a disturbed digraph. Here, the Aho graph *H* was connected in 60% of the initial sample. Thus, even moderate perturbation of a BMG introduces inconsistencies into the triple set ${\mathcal {R}}(\vec {G},\sigma )$ and results in a connected Aho graph *H* in the majority of cases. As shown in Fig. 2, both arc insertions and deletions can cause triple inconsistencies. For better comparison, the same set of test instances is used for all of the methods described below.

### Heuristics for (B)PURC

(B)PURC is a variation on graph partitioning problems. It seems reasonable, therefore, to adapt graph partitioning algorithms for our purposes.

*MinCut.* We solve the minimum edge cut problem for the connected undirected graph *H*, i.e., we want to find a bipartition ${\mathcal {V}}=\{V_1,V_2\}$ such that the number of edges between $V_1$ and $V_2$ is minimal in *H*. The problem can be solved exactly in polynomial time using the Stoer-Wagner algorithm [23]. Note, however, that the minimum edge cut in *H* will in general not deliver an optimal solution of (B)PURC.

*Karger’s algorithm* is a randomized algorithm that, in its original form, also aims to find a minimum edge cut [24]. In brief, it merges vertices of the graph by randomly choosing and contracting edges, until only two vertices remain, which induce a bipartition ${\mathcal {V}}$ according to the vertices that were merged into them. By repeating this process a sufficient number of times, a minimum edge cut can be found with high probability. Here, we use the *UR*-cost $c(\vec {G},{\mathcal {V}})$ instead of the size of the edge cut as objective function to select the best solution over multiple runs.

*A simple greedy approach* starts with ${\mathcal {V}}=\{V_1=\emptyset , V_2=V'\}$ and stepwise moves a vertex $v\in V_2$ to $V_1$ such that $c(\vec {G},\{V_1\cup \{v\},V_2\setminus \{v\}\})$ is optimized. Ties are broken at random. This produces $|V|-1$ “locally optimal” bipartitions, from which the best one is selected.

*Gradient walks.* The space of all bipartitions ${\mathcal {V}}=\{V_1,V_2\}$ endowed with a “move set” and the objective function $c(\vec {G},{\mathcal {V}})$ forms a fitness landscape. Here, we consider adjacency between bipartitions by moving one vertex from $V_1$ to $V_2$ or *vice versa*. Gradient walks [25], also called “gradient adaptive walk” [26] or “greedy adaptive walks” [27], form the discrete analog of gradient descent methods. We start with a random but balanced bipartition ${\mathcal {V}}=\{V_1, V_2\}$ and then repeatedly execute a move to an adjacent bipartition that maximally improves the objective function; a gradient walk stops when a local optimum is reached.

*Louvain method.* This method for community detection in graphs greedily optimizes the so-called modularity of a vertex partition ${\mathcal {V}}$ [28]. Its objective function is $q({\mathcal {V}}) = \sum _{W\in {\mathcal {V}}}\sum _{u,v\in W} (a_{uv}-d_ud_v/(2m))$, where $a_{uv}$ are the entries of the (possibly weighted) adjacency matrix of a graph *H*, $d_u=\sum _v a_{uv}$ the vertex degrees, and *m* is the sum of all edge weights in the graph. This favors so-called *communities* or *modules* *W* that are highly connected internally but have only few edges between them. The Louvain method operates in two phases starting from the discrete partition ${\mathcal {V}}=\{\{u\}\,|\, u\in V\}$. In the first phase, it repeatedly iterates over all vertices *x* and moves *x* into the community of one of its neighbors that leads to the highest gain in modularity as long as a move that increases $q({\mathcal {V}})$ can be found. The second phase repeats the first one on the weighted quotient graph $H/{\mathcal {V}}$ whose vertices are the sets of ${\mathcal {V}}$ and whose edge weights are the sum of the original weights between the communities. In addition to maximizing the modularity, we also investigate a variant of the Louvain method that moves vertices into the community of one of their neighbors if this results in a lower *UR*-cost $c(\vec {G},{\mathcal {V}})$, and otherwise proceeds analogously. We exclude the merging of the last two vertices to ensure that a non-trivial partition is returned. Since the Louvain method is sensitive to the order in which the vertex set is traversed, we randomly permute the order of vertices to allow multiple runs on the same input.

With the exception of the Stoer-Wagner algorithm for solving the minimum edge cut problem, all of these partitioning methods include random decisions. One may therefore run them multiple times and use the partition corresponding to the best objective value, i.e., the lowest *UR*-cost $c(\vec {G},{\mathcal {V}})$ or the highest modularity. If not stated otherwise, we apply five runs for each of these methods in each recursion step (with a connected Aho graph) in the following analyses.

### Heuristics for BMG editing

We will explore the performance of several variants of Alg. 1 and 2 for BMG editing. The variants of Alg. 2 correspond to using the heuristics for (B)PURC discussed above for processing a connected Aho graph $H{:}{=}[{\mathcal {R}}(\vec {G}^*[V'],\sigma _{|V'}),V']$ for the triples ${\mathcal {R}}(\vec {G}^*[V'],\sigma _{|V'})$ in each step of the recursion. We note that Alg. 2 in combination with any of the heuristics for (B)PURC also serves as a heuristic for MaxRTC because the choice of the partition ${\mathcal {V}}$ in each recursion step determines a set of included triples *xy*|*z*, namely those for which *x* and *y* are contained in one set of ${\mathcal {V}}$ while *z* is contained in another. Another way of expressing that same fact is that an approximation to MaxRTC is given by the subset ${\mathcal {R}}^*\subseteq {\mathcal {R}}(\vec {G},\sigma )$ of the informative triples of the input digraph $(\vec {G},\sigma )$ that are displayed by the tree *T* constructed in Alg. 2. In particular, Alg. 2 together with the *MinCut* method has been described as a heuristic for MaxRTC in earlier work [16, 17]. For comparison, we will also consider the following bottom-up approach as a component of Alg. 1:

*Best-Pair-Merge-First (BPMF)* was described in [18], and constructs a tree from a set of triples ${\mathcal {R}}$ in a bottom-up fashion. We use here a modified version introduced in [16]. BPMF operates similar to the well-known UPGMA clustering algorithm [29]. Starting with each vertex $x\in V$ as its own cluster, pairs of clusters are merged iteratively, thereby defining a rooted binary tree with leaf set *V*. The choice of the two clusters to merge depends on a similarity score with the property that any triple *xy*|*z* with *x*, *y*, and *z* lying in distinct clusters $S_x$, $S_y$, and $S_z$ contributes positively to $\text {score}(S_x,S_y)$ and negatively to $\text {score}(S_x,S_z)$ and $\text {score}(S_y,S_z)$. Since BPMF constructs the tree *T* from the bottom, it does not imply a vertex partitioning scheme that could be plugged into the top-down procedure of Alg. 2. Importantly, BPMF is not a consistent heuristic for MaxRTC, i.e. it does not necessarily recognize consistent triples sets. Hence, consistency in the application to BMG editing is also not guaranteed, see Fig. 15 in Appendix B for an example.

In summary, we have two distinct ways to obtain an edited BMG: We may take either

1. $\vec {G}(T,\sigma )$, where *T* is the output tree of Alg. 2 or BPMF, respectively, or
2. $\vec {G}(T^*, \sigma )$, where $T^*={{\,\mathrm{Aho}\,}}({\mathcal {R}}^*,V(\vec {G}))$ is constructed from the consistent triple subset of triples ${\mathcal {R}}^*$. This corresponds to Alg. 1.

Somewhat surprisingly, the results in Fig. 7 below suggest that it is in general beneficial to extract the triple set ${\mathcal {R}}^*$ and rerun the BUILD algorithm, i.e., to use $\vec {G}(T^*,\sigma )$.

### Heuristics for binary-explainable BMG Editing

In order to test the heuristics for the slightly different task of obtaining a binary-explainable BMG $(\vec {G}^*,\sigma )$, we constructed a similar set of test instances. The only difference is that we ensured that $T_\text {orig}$ is binary by modifying the attachment procedure above such that in each growth step we only choose among the vertices that are currently leaves for attaching two new leaves. Thus, $(\vec {G}_\text {orig},\sigma )=\vec {G}(T_\text {orig},\sigma )$ is binary-explainable. The editing heuristics are analogous, with two straightforward modifications:

- In the Aho graphs, $\mathop {{\mathcal {R}}^{\text {B}}}(\vec {G},\sigma )$ is used instead of ${\mathcal {R}}(\vec {G},\sigma )$.
- If we encounter a partition ${\mathcal {V}}$ of cardinality greater than two in some recursion step, we use a coarse-graining ${\mathcal {V}}'$ of ${\mathcal {V}}$ such that $|{\mathcal {V}}'|=2$ instead. This modification is necessary whenever $[\mathop {{\mathcal {R}}^{\text {B}}}(\vec {G},\sigma )[V'], V']$ itself has more than two connected components, and for the partitions with $|{\mathcal {V}}|\ge 3$ returned by the Louvain method.

By Thm. 29, this procedure is consistent for binary-explainable BMGs. Thm. 29, moreover, guarantees some freedom in the choice of a coarse-graining ${\mathcal {V}}'=\{V_1, V_2\}$ whenever ${\mathcal {V}}$ is not a bipartition. We therefore aim to produce (locally) balanced trees in such situations, i.e., we seek to minimize the difference of $|V_1|$ and $|V_2|$. Formally, this corresponds to the well-known Number Partitioning problem with the multiset $\{|V_i| \,\mid \, V_i \in {\mathcal {V}}\}$ as input. We use the efficient heuristic described in [30], which in general appears to yield very good solutions of the Number Partitioning problem [31].

To construct the second binary tree $T^*$ based on subset of triples ${\mathcal {R}}^*\subseteq \mathop {{\mathcal {R}}^{\text {B}}}(\vec {G},\sigma )$ that are displayed by *T*, we employ an analogous coarse-graining in an otherwise unmodified BUILD algorithm. We note, however, that one could incorporate more sophisticated approaches which e.g. use some greedy coarse-graining method based on the *UR*-cost.

## Computational results

In this section, we compare different heuristics for the (B)PURC Problem and their performance in the context of BMG editing. Somewhat unexpectedly, but in accordance with Fig. 5, our results suggest that a good (or bad) performance of (B)PURC is not directly linked to a good (or bad) performance for BMG editing. Moreover, we find that, even for noisy data, all analyzed methods are able to capture the tree structure of the underlying “true” BMG at least to some extent. As we shall see, community detection approaches in combination with the *UR*-cost appear to be more promising for BMG editing than optimal solutions of (B)PURC alone.

**Fig. 6:**
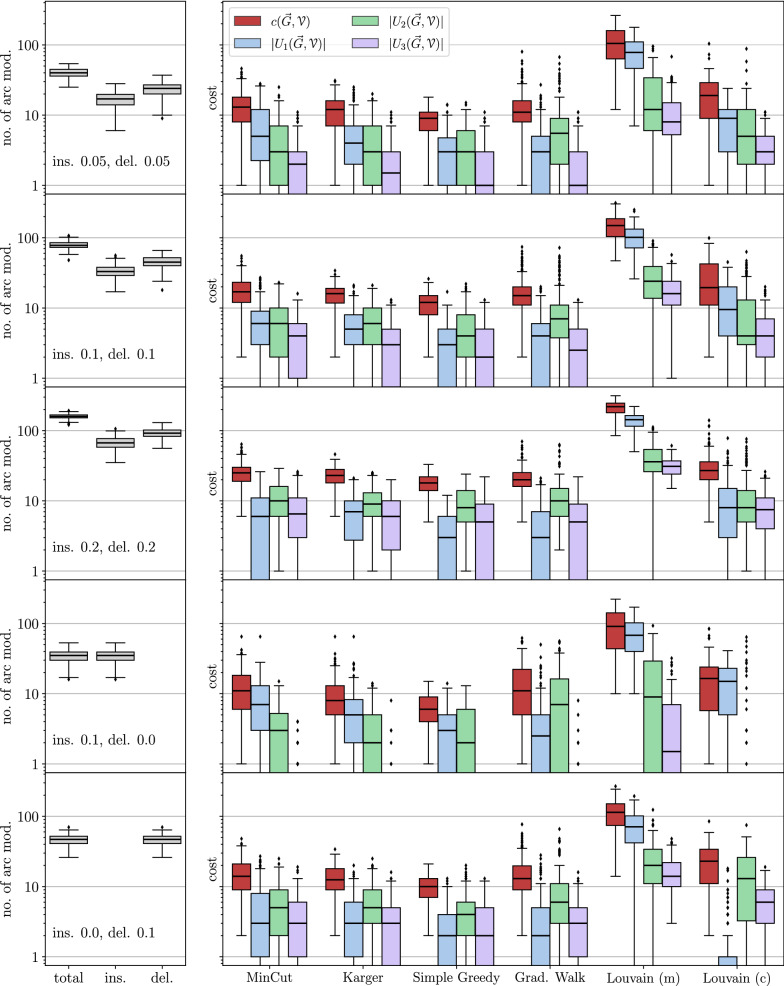
Performance of partitioning methods for minimizing $c(\vec {G},{\mathcal {V}})$ on perturbed BMGs $(\vec {G},\sigma )$. The rows correspond to different insertion and deletion probabilities (indicated in the l.h.s. panels) used to disturb the original BMGs. The l.h.s. panels show the distribution of the no. of arc modifications in total, arc insertions and arc deletions of the disturbed digraphs w.r.t. the original BMGs. The r.h.s. panels show the distribution of *UR*-costs $c(\vec {G},{\mathcal {V}})$ (red) obtained for each method, and of the no. of arcs in $U_1(\vec {G},{\mathcal {V}})$, $U_2(\vec {G},{\mathcal {V}})$, and $U_3(\vec {G},{\mathcal {V}})$ (i.e., the sets that contribute to the *UR*-cost). Example plot for $|V|=30$ vertices and $|\sigma (V)|=10$ colors in each digraph. Among the 200 generated digraphs, only those with a connected Aho graph $[{\mathcal {R}}(\vec {G},\sigma ), V(\vec {G})]$ are included in each of the five rows (93%, 100%, 100%, 60%, 95%)

In order to better understand the behavior of the repeated application of the partitioning heuristics of Alg. 2, it is instructive to consider not only the score but also the structure of partitions. We observe a strong tendency of some of the partitioning methods to produce *single-leaf splits*, i.e., (bi)partitions ${\mathcal {V}}$ in which at least one set $W\in {\mathcal {V}}$ is a singleton (i.e., $|W|=1$). Single-leaf splits in general seem to have relatively low *UR*-costs. Further details on the propensity of the partitioning heuristics to produce single-leaf splits are given in Appendix C.

### Results for (B)PURC

Fig. 6 suggests that the *Simple Greedy* approach is best suitable for the minimization of the *UR*-cost $c(\vec {G},{\mathcal {V}})$ for any of the considered parameters for BMG disturbance. The Louvain method based on graph modularity (*Louvain (m)*) appears to have by far the worst performance which, moreover, quickly produces higher *UR*-costs with an increasing intensity of the perturbations.

### Results for BMG editing

**Fig. 7:**
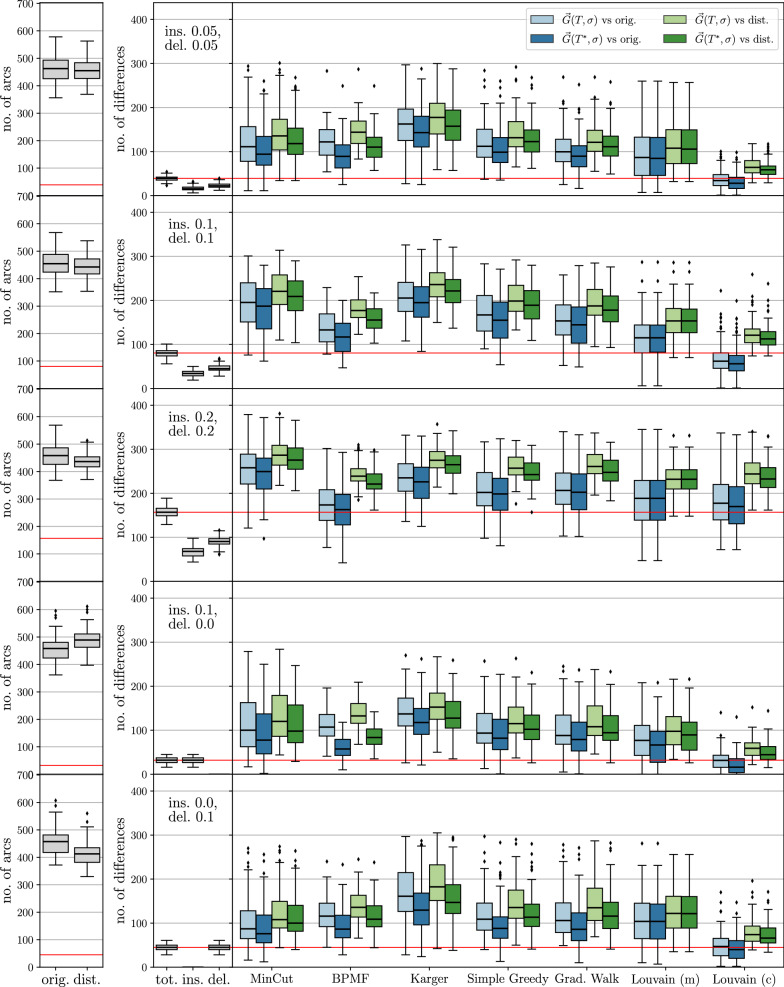
Performance comparison of several BMG editing heuristics based on the no. of arc differences. The rows correspond to different insertion and deletion probabilities (indicated in the second column panels) used to perturb the original BMGs. The l.h.s. panels show the distribution of the no. of arcs in the original BMG and in the perturbed digraph. The second column panels show the distribution of the no. of arc modifications in total, arc insertions and arc deletions of the perturbed digraphs w.r.t. the original BMGs. The red lines mark the median values of the total no. of modifications. The r.h.s. panels show the total no. of arc differences w.r.t. the original random BMGs (blue) and the perturbed digraphs (green). The light colors indicate the “direct” performance of each method, i.e., the digraph $\vec {G}(T,\sigma )$ where *T* is the tree that is directly constructed by each method. The darker colors indicate the results if the methods are used as heuristic for MaxRTC in Alg. 1. Example plot for $|V|=30$ vertices and $|\sigma (V)|=10$ colors in each digraph, 100 digraphs per row

**Fig. 8:**
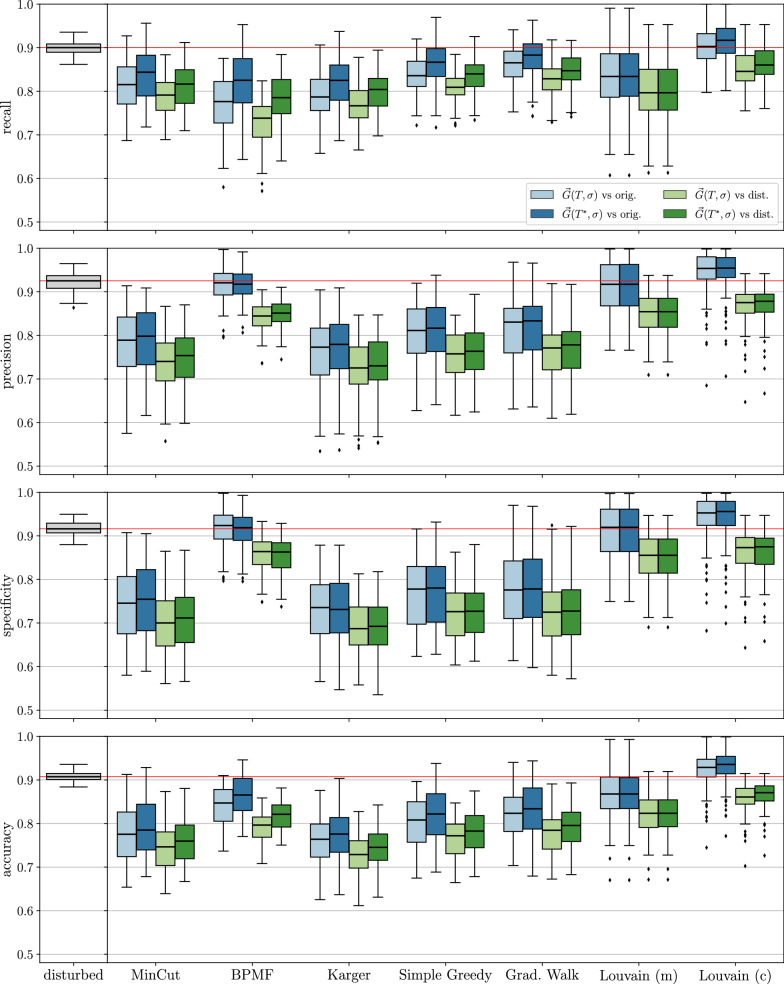
Performance comparison of several BMG editing heuristics based on recall, precision, specificity, and accuracy (rows 1 to 4). The l.h.s. panels show the respective measure for the perturbed digraph w.r.t. the original random BMG. The red lines marks the median values of the latter. The r.h.s. panels show the results for the edited digraphs w.r.t. the original BMGs (blue) and the perturbed digraphs (green). The light colors indicate the “direct” performance of each method, i.e., the digraph $\vec {G}(T,\sigma )$ where *T* is the tree that is directly constructed by each method. The darker colors indicate the results if the methods are used as heuristic for MaxRTC in Alg. 1. Example plot for $|V|=30$ vertices and $|\sigma (V)|=10$ colors in each digraph, insertion and deletion probability 0.1, and 100 digraphs

To assess the performance of the various heuristics for BMG editing, we consider the differences between the editing result $(G^*,\sigma )$ from both the original BMG $(G_{orig},\sigma )$ and the perturbed input digraphs $(G,\sigma )$. In Fig. 7, we summarize the absolute values of the symmetric differences of the arc sets $d_{orig} {:}{=}|E(G^*){{\,\mathrm{\triangle }\,}}E(G_{orig})|$ and $d {:}{=}|E(G^*){{\,\mathrm{\triangle }\,}}E(G)|$, respectively. These results are translated to usual normalized performance indicators (recall, precision, specificity, and accuracy; all defined in terms of the arc sets) in Fig. 8.

Comparing the distances $d_{orig}$ (blue boxplots) and *d* (green boxplots) of the editing result $(G^*,\sigma )$ to original unperturbed BMG and the input digraph, resp., we find that, for the methods investigated here, on average $d_{orig}$ is smaller than *d*. This indicates that all methods are able to capture the underlying tree structure of the original BMG at least to some extent. The discrepancy between $d_{orig}$ and *d* tends to increase with the level of perturbation, a trend that is most pronounced for *Louvain (c)*. This result is encouraging for practical applications of BMG modification to correcting noisy best match data, where the eventual goal is to obtain a good estimate of the underlying true BMG.

Intriguingly, the extraction of consistent informative triples ${\mathcal {R}}^*$ from the reconstructed tree *T* and rerunning BUILD, i.e., using $\vec {G}(T^*,\sigma )$, in general improves the estimation results for the majority of methods. In particular, this increases the recall without a notable negative impact on precision and specificity (cf. Fig. 8). A better recall, corresponding to a higher proportion of correctly inferred arcs, is not surprising in this context, since this additional step in essence reduces the number of triples. We therefore expect the tree $T^*={{\,\mathrm{Aho}\,}}({\mathcal {R}}^*,V(\vec {G}))$ to be on average less resolved than *T*. The BMGs of less resolved trees tend to have more arcs than BMGs of highly resolved tree (cf. [12, Lemma 8]). In good accordance with this prediction, *BPMF*, which shows a strong increase of recall, always constructs a binary, i.e., fully-resolved, tree *T* – whereas the corresponding tree $T^*$ in general is much less resolved.

Somewhat surprisingly, a good or bad performance for minimizing the *UR*-cost in individual steps apparently does not directly translate to the performance in the overall editing procedure. In particular, the modularity-based *Louvain (m)* method seems to be a better choice than the *Simple Greedy* approach. The methods *MinCut* and *Karger* do not seem to be suitable components for Alg. 2, with the exception of the case where perturbations are arc deletions only (Fig. 7, bottom row). Here, *MinCut* produces reasonable estimates that compare well with other methods. The bottom-up method for the MaxRTC problem *BPMF* also produces relatively good results. It appears to be robust at high levels of perturbation. For most of the parameter combinations, we obtain the best results with the *UR*-cost-based Louvain method (*Louvain (c)*). Here, we often observe a symmetric difference (w.r.t. the arcs sets) that is better than the difference between the original and the perturbed digraph. This trend is illustrated by the red median lines in Fig. 7 and 8 . Hence, we achieve two goals of BMG editing: (i) the resulting digraph $(\vec {G}^*,\sigma )$ is a BMG, i.e., it satisfies Def. 1, and (ii) it is closer to the original BMG than the perturbed digraph. We note that we observed similar trends across all investigated combinations for the numbers of leaves *N* (ranging from 10 to 40) and of colors $\ell$ ($\ell <N$ ranging from 2 to at most 20).

**Fig. 9:**
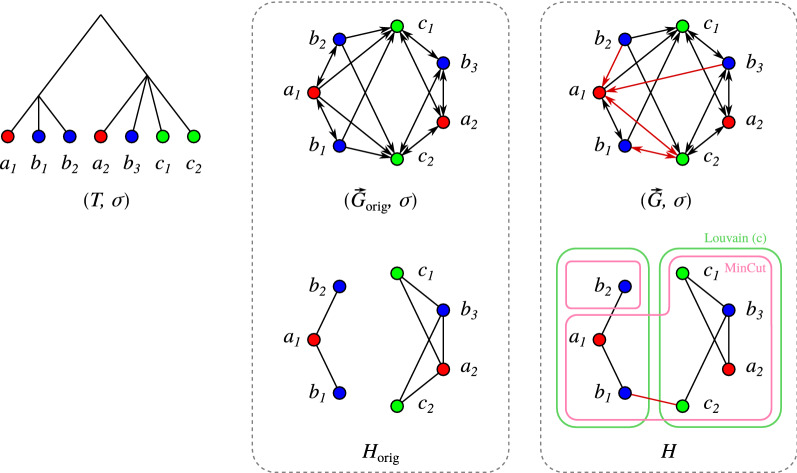
Example of an instance where the Louvain method performs better due to more balanced partitions. The (least resolved) tree $(T,\sigma )$ explains the BMG $(\vec {G}_\text {orig},\sigma )$ with vertex set *V*. The graph $H_\text {orig}=[{\mathcal {R}}(\vec {G}_\text {orig},\sigma ), V]$ is the Aho graph corresponding to the informative triple set ${\mathcal {R}}(\vec {G}_\text {orig},\sigma )$. The perturbed digraph $(\vec {G},\sigma )$ is obtained from $(\vec {G}_\text {orig},\sigma )$ by inserting the arcs $(b_3,a_1)$, $(c_2,a_1)$, and $(c_2, b_1)$ and deletion of $(a_1,b_2)$. The corresponding Aho graph $H=[{\mathcal {R}}(\vec {G},\sigma ), V]$ is connected because the perturbation introduced the additional informative triple $c_2b_1|b_2$. The green and pink frames correspond to the partitions ${\mathcal {V}}_1$ and ${\mathcal {V}}_2$ of *V* constructed by the methods *Louvain (c)* and *MinCut*, respectively

Our results show that minimization of the *UR*-cost in each step is not the best approach to BMG editing because this often produces very unbalanced partitions. As a consequence, more recursion steps are needed in Alg. 2 resulting in higher accumulated number of arc edits. Figure 9 shows that better solutions to the BMG editing problem are not necessarily composed of vertex partitions with minimal *UR*-cost in each step. The perturbed digraph $(\vec {G},\sigma )$ in Fig. 9 was obtained from the randomly simulated BMG $(\vec {G}_\text {orig},\sigma )$ as described above using equal insertion and deletion probabilities of 0.1. As an example, the partitions ${\mathcal {V}}_1$ and ${\mathcal {V}}_2$ as constructed by the *MinCut* and the *Louvain (c)* method in the first iteration step of Alg. 2 are shown as pink and green frames, respectively. *MinCut* produces a single-leaf split ${\mathcal {V}}_1$ with an isolated vertex $b_2$ and *UR*-cost $c(\vec {G},{\mathcal {V}}_1)=1$ deriving from $U_1(\vec {G},{\mathcal {V}}_1)=\{(b_2,a_2)\}$. *Louvain (c)* identifies the partition ${\mathcal {V}}_2$ with $c(\vec {G},{\mathcal {V}}_2)=3$ originating from $U_2(\vec {G},{\mathcal {V}}_1)=\{(b_3,a_1),(c_2,a_1), (c_2,b_1)\}$, which corresponds to the connected components of the Aho graph $H_\text {orig}$ of the unperturbed BMG and thus identifies the split in the original tree $(T,\sigma )$. Here, the correct partition ${\mathcal {V}}_2$ has a strictly larger *UR*-cost than the misleading choice of ${\mathcal {V}}_1$. However, *MinCut* results in a higher total edit cost than *Louvain (c)* for $(\vec {G},\sigma )$.

In order to account for the issue of unbalanced partitions, we performed a cursory analysis on maximizing a gain function rather than minimizing the *UR*-cost. In analogy to $c(\vec {G},{\mathcal {V}})$, we defined $g(\vec {G},{\mathcal {V}})$ as the number of arcs and non-arcs that are satisfied by the BMGs of *all* trees in ${\mathcal {T}}({\mathcal {V}})$. Recapitulating the arguments in the proof of Lemma 13, one can show that these relations can also be determined as the union of three sets by replacing “$(x,y)\in E$” with “$(x,y)\notin E$” and *vice versa* in the definitions of $U_1(\vec {G},{\mathcal {V}})$, $U_2(\vec {G},{\mathcal {V}})$, and $U_3(\vec {G},{\mathcal {V}})$. The gain function $g(\vec {G},{\mathcal {V}})$ can be used instead of the *UR*-cost with *Karger*, *Simple Greedy*, *Gradient Walk*, and in a gain-function-based *Louvain* method. For all these algorithms, however, maximizing $g(\vec {G},{\mathcal {V}})$ leads to partitions that appear to be *too* balanced, and a performance for BMG editing that is worse than the use of the *UR*-cost. A possible explanation for both unbalanced and too balanced partitions as produced with a cost and gain function, resp., is the fact that $U_1(\vec {G},{\mathcal {V}})$ and $U_2(\vec {G},{\mathcal {V}})$ (and their gain function counterparts) contain pairs of vertices (*x*, *y*) that lie in distinct sets of ${\mathcal {V}}$. Hence, both single-leaf splits and perfectly balanced partitions minimize (maximize, resp.) the number of pairs that could potentially be contained in these arc sets.

**Fig. 10:**
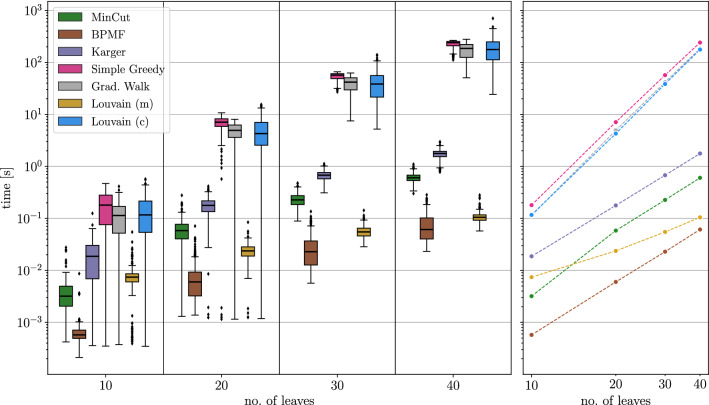
Running times of the different methods for BMG editing. The time only includes the construction of the tree *T*, i.e., Alg. 2 or *BPMF*, resp., but not the extraction of the triple set ${\mathcal {R}}^*$ followed by rerunning BUILD. For each number of leaves $N\in \{10,20,30,40\}$ and each number of colors $\ell$ (taken from $\{2,5,10,20\}$ such that $\ell <N$), 100 perturbed BMGs were generated using equal insertion and deletion probabilities of 0.1. In the right panel, the median values are shown with logarithmic axes

All methods for BMG editing were implemented and compared using Python on an off-the-shelf laptop (Intel$^\circledR$ ${\text {Core}}^{\text{TM}}$ i7-4702MQ processor, 16 GB RAM, Ubuntu 20.04, Python 3.7). They are available as a Python library at https://github.com/david-schaller/bmg-edit. Figure 10 summarizes the running times. The right panel shows that all methods appear to scale polynomially in the size |*V*| of the vertex set of the input digraph. The methods that explicitly rely on the *UR*-cost are much slower than the other methods. We suspect that this is largely due to the repeated $O(|V'|^2)$-computation of $c(\vec {G},{\mathcal {V}})$ whenever a vertex is moved between the sets/communities in ${\mathcal {V}}$. This could possibly be improved by an incremental algorithm.

### Results for binary-explainable BMG Editing

**Fig. 11:**
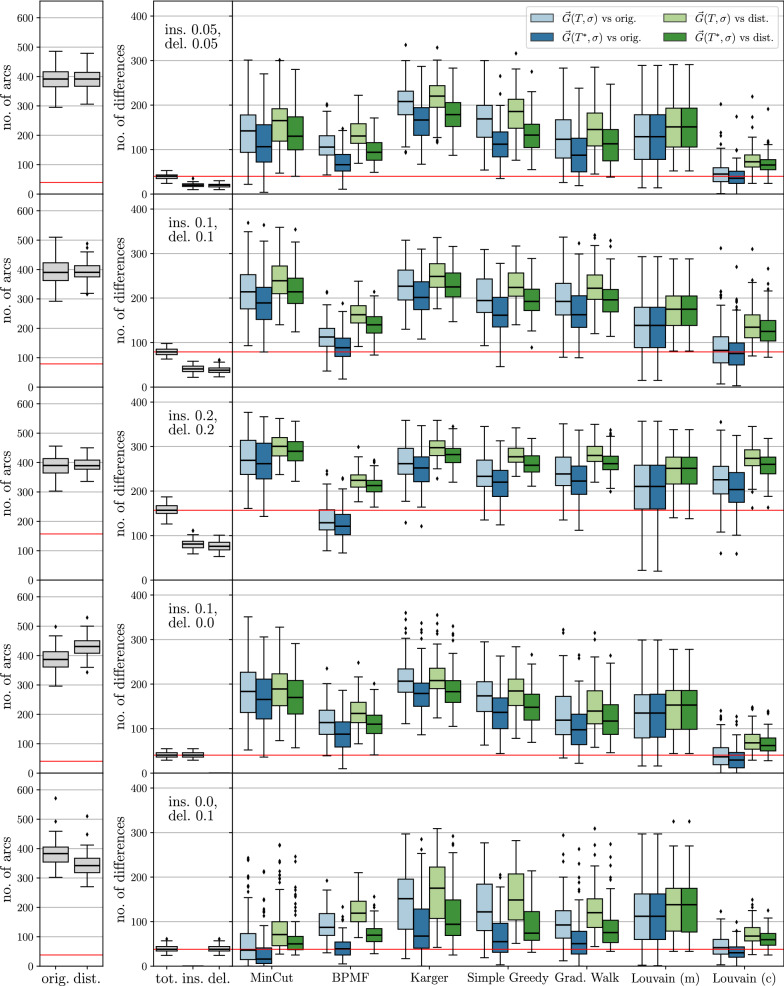
Performance comparison of several beBMG editing heuristics based on the no. of arc differences. See Fig. 7 for further description. Example plot for $|V|=30$ vertices and $|\sigma (V)|=10$ colors in each digraph, 100 digraphs per row

The results for beBMG editing in essence recapitulate the observations for general BMG editing, see Fig. 11. Alg. 2 in combination with *Louvain (c)* appears to be the best choice for the majority of parameter combinations. However, it is outperformed by the *BPMF* heuristic at high levels of perturbation (insertion and deletion probability 0.2). As in the general case, construction of $T^*$ and using $(\vec {G}^*,\sigma ){:}{=}\vec {G}(T^*,\sigma )$ as editing result appears to be advantageous. Moreover, the difference of the editing result $(\vec {G}^*,\sigma )$ to the original beBMG $(\vec {G}_\text {orig},\sigma )$ is on average smaller than the difference of $(\vec {G}^*,\sigma )$ to the perturbed digraph $(\vec {G},\sigma )$.

### Real-life data

Assessing the performance of BMG editing for real-life data is not a trivial task because no reliable gold standard data sets are available. Moreover, we expect that software pipelines for best match inference would benefit from a pre-processing step that eliminates systematic errors arising as a consequence of unequal evolution rates in different branches of a gene family [5]. The implementation of such a pipeline is beyond the scope of this contribution. Nevertheless, we include a brief analysis of a small set of eubacterial genomes to obtain a first impression of the practical applicability of the methods described in this contribution. In particular, we provide an empirical justification for the level of error introduced in the simulated digraphs.

We consider the genomes of the eleven species of Aquificales species that have been studied previously in [32]. Starting from the protein sequences of these species, we use ProteinOrtho [33] to obtain estimates for best matches. ProteinOrtho is a tool for orthology inference that, in a first step, constructs a digraph ${\Upsilon }$ on the set of genes/proteins from all species. The digraph contains an arc (*x*, *y*) whenever *x* and *y* stem from distinct species *X* and *Y*, respectively, and the sequence of *y* is among the most similar sequences in *Y* to that of *x*. Sequence similarity is measured here in terms of bitscores obtained from the hits in an all-*versus*-all comparison of the sequences using blast or a fast local alignment tool. Moreover, cutoffs for the E-value and the sequence identify are set to avoid an overabundance of spurious hits. Here, we use the default settings of the current ProteinOrtho version, i.e., DIAMOND [34] is employed for the all-*versus*-all comparison and we only include hits with an E-value smaller than $10^{-5}$ and a pairwise sequence identity exceeding 25%. To better account for the fact that multiple best matches of *x* in species *Y* are possible, we choose a more inclusive relative threshold $f=0.8$ for the bitscores (as compared to the default value $f=0.95$), i.e., an arc (*x*, *y*) is included in ${\Upsilon }$ whenever the respective hit reaches a bitscore of at least 0.8 times the bitscore of the best hit of *x* in species *Y*. To infer orthologous genes, i.e., related genes that arose from speciation events, ProteinOrtho proceeds to construct the symmetric part $\Upsilon$ of ${\Upsilon }$ followed by spectral clustering to eliminate false orthology edges [33]. Since we are interested in the directed best match graph rather than orthology, we use ${\Upsilon }$ as an estimate of the best match relation and forego the rest of the pipeline.

**Fig. 12:**
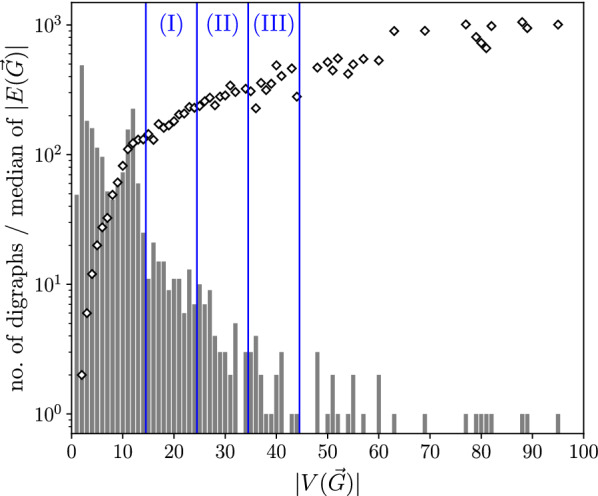
Summary of the data set of protein-coding gene families from 11 genomes of Aquificales, see [35] for details. We show the distribution of the number of vertex-colored digraphs $(\vec {G},\sigma )$ (histogram) and the median number of arcs $|E(\vec {G})|$ ($\diamond$ symbols) for the estimated BMGs as a function of the number of genes $V|(\vec {G})|$ in each family. The bins (I) $15\le |V(\vec {G})|< 25$, (II) $25\le |V(\vec {G})|< 35$, and (III) $35\le |V(\vec {G})|< 45$ are used for the comparison of the BMG editing methods, see text for details

The resulting digraph ${\Upsilon }$ for the Aquificales data set comprises 16630 vertices and 2001 (weakly) connected components. The distribution of the order of these components is shown in Fig. 12. We obtained 9 components with more than 100 vertices (with a maximum of 775 vertices), which are not included in the plot. We use each connected component $(\vec {G},\sigma )$ of ${\Upsilon }$, where $\sigma$ is determined by the species to which a gene/protein belongs, as input for our editing heuristics. We distinguish three size classes (bins) as indicated in Fig. 12 to discuss the results. It is worth noting that in particular the large components may still be composites of genes that are not true homologs but only share certain protein domains. For routine applications, additional data preprocessing steps are advisable. Gene family classification, i.e., the clustering of proteins in families [36], is itself by no means a completely solved problem in computational biology.

**Fig. 13:**
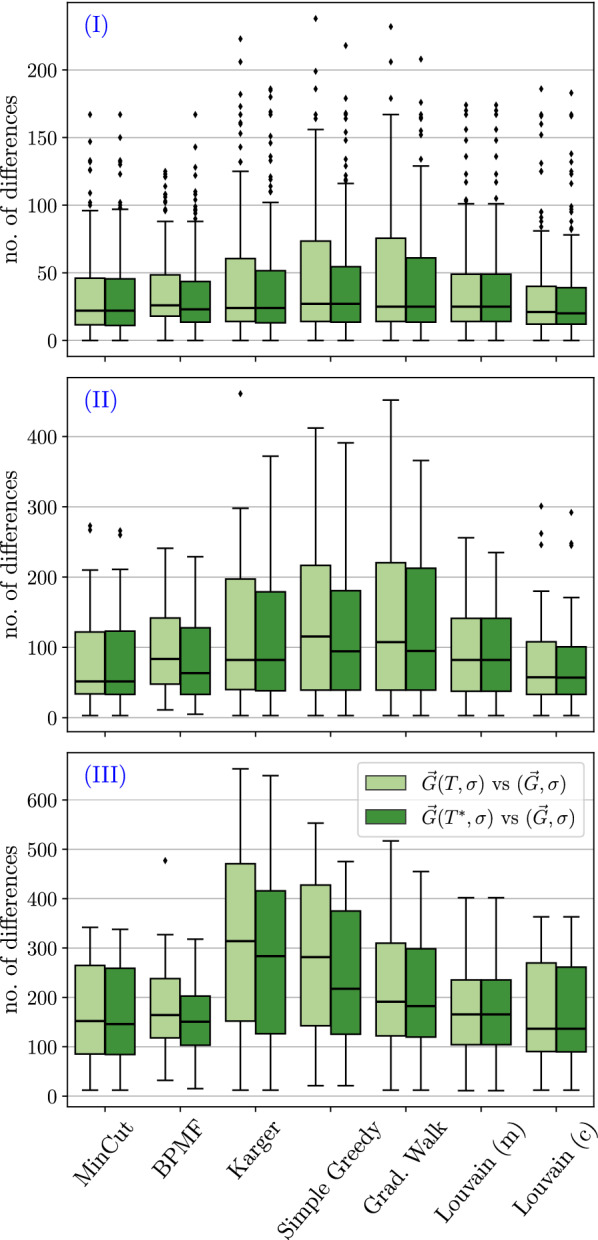
Performance of the BMG editing heuristics for the Aquificales data, measured as the number of arc differences between input digraphs and editing results. As above, the light green indicates the “direct” performance of each method, i.e., the digraph $\vec {G}(T,\sigma )$ where *T* is the tree that is directly constructed by each method, whereas the dark green indicates the results if the methods are used as heuristic for MaxRTC in Alg. 1. The three panels show the results for the bins (I) $15\le |V(\vec {G})|< 25$, (II) $25\le |V(\vec {G})|< 35$, and (III) $35\le |V(\vec {G})|< 45$, respectively, as indicated in Fig. 12

Figure 13 summarizes the number of arc differences between the editing results and the input digraphs. As in the simulations, the input digraphs are in general not valid BMGs. Indeed, we found that only 5.9% in bin (I) comprising small instances and none of the digraphs in bins (II) and (III) were BMGs. The comparison of the different methods recapitulates the results on simulated data. Differences in the performance are on average more prominent for input digraphs with more vertices. The methods *BPMF* and *Louvain (c)* again show good results. However, here, the simple *MinCut* heuristic is also among the best-performing methods. As before, the editing can be slightly improved by additionally constructing $T^*$ and its BMG $\vec {G}(T^*,\sigma )$. Comparing e.g. the top panel in Fig. 7 ($|V(\vec {G})|=30$ for all digraphs) and bin (II) in Fig. 13 shows that the number of arc edits performed by the heuristics is comparable for simulated and real-life data. Even though the true BMG and thus the amount of errors is not known in the Aquificales data set, this suggests that simulation results provide a realistic view of BMG editing in real-life applications.

## Summary and discussion

In this contribution, we have described a large class of heuristics for BMG editing that operate in a recursive top-down fashion to (at least implicitly) construct a tree $(T,\sigma )$ capturing the underlying BMG-structure of an arbitrary input digraph $(\vec {G},\sigma )$. We have shown that this is closely related to a specific notion of locally good edits, which we assess using the *UR*-cost. The *UR*-cost counts the minimum number of arc insertions and deletions of the BMG-editing for $(\vec {G},\sigma )$ that are linked to each inner node (and thus to their corresponding leaf partitions) in $(T,\sigma )$ and cannot be reversed in subsequent recursion steps. In particular, we showed that an optimal solution among all possible partitions guarantees consistency of this class of heuristics (cf. Thm. 23 and 29). Unfortunately, the corresponding problem BPURC is itself NP-complete.

We therefore suggested a number of approximation methods for finding suitable partitions, and compared their performances in the context of Alg. 2. We find that, even though good solutions for (B)PURC alone do not seem to be the most adequate approach, the value of the *UR*-costs appears most clearly in a combination with a method for community detection, more precisely, a modification of the Louvain method [28].

For all of the methods investigated here, we found that the Aho graph $H{:}{=}[{\mathcal {R}}(\vec {G},\sigma )[V'], V']$ serves as a useful starting point for finding a suitable partition. This choice is based on the idea that, due to the properties of BMGs and in particular the construction of the tree $(T,\sigma )$ from informative triples of the BMG $(\vec {G},\sigma )=\vec {G}(T,\sigma )$, arc insertions and deletions in $(\vec {G},\sigma )$ should not add too many new edges between the connected components of the originally disconnected Aho graph of ${\mathcal {R}}(\vec {G},\sigma )$ (cf. Fig. 2). Therefore, we suggest that there is a correlation between good partitions ${\mathcal {V}}$ of $V'$, i.e. partitions linked to few edits, and the minimization of the number of edges in *H* connecting vertices in distinct sets of ${\mathcal {V}}$.

For the general BMG editing problem, we did not make use of the information contained in the set of forbidden triples ${\mathcal {F}}(\vec {G},\sigma )$ of the input digraph $(\vec {G},\sigma )$. It might be possible to adapt the algorithm MTT [13], which identifies consistent pairs $({\mathcal {R}},{\mathcal {F}})$, instead of BUILD. MTT constructs a coarse-graining ${\mathcal {V}}_\text {MTT}$ of the set of connected components of the Aho graph (on ${\mathcal {R}}$) in order to account for the forbidden triples in ${\mathcal {F}}$ in each recursion step. Possibly, ${\mathcal {V}}_\text {MTT}$ (or some suitable graph representation) yields a further improvement. However, in case of beBMG editing, the extended triple set $\mathop {{\mathcal {R}}^{\text {B}}}(\vec {G},\sigma )$ and thus the corresponding Aho graphs by construction already cover the information contained in ${\mathcal {F}}(\vec {G},\sigma )$. Since no substantial improvement over the general case was observed in this case (cf. Fig. 11), we opted against more detailed benchmarking of ${\mathcal {V}}_\text {MTT}$ in comparison to partitions based on the Aho graph.

Another source of information that we have not considered here are values of confidence for best matches e.g. derived from pairwise sequence alignment. These can naturally be provided as weights assigned to the arcs of an input digraph $(\vec {G}=(V,E),\sigma )$. Since every informative triple $ab|b'\in {\mathcal {R}}(\vec {G},\sigma )$ stems from an arc $(a,b)\in E$ (and a “non-arc” $(a,b')\notin E$), such arc weights can be propagated to the triples in ${\mathcal {R}}(\vec {G},\sigma )$. Assuming that larger weights mean higher confidence, a simple approach to weighting the edges *xy* in an Aho graph $H{:}{=}[{\mathcal {R}}(\vec {G},\sigma )[V'], V']$ is to sum up the weights of all triples $xy|z\in {\mathcal {R}}(\vec {G},\sigma )[V']$ that induce the edge $xy\in E(H)$. Now the heuristics for finding a (bi)partition ${\mathcal {V}}$ in Alg. 2 may operate on this weighted graph *H*. Methods such as the Stoer-Wagner algorithm [23] and the (modularity-based) Louvain method [28] natively support weighted graphs. If the *UR* are used to find ${\mathcal {V}}$, the situation appears more complicated. The cost contributed by an arc $(x,y)\in U_1(\vec {G},{\mathcal {V}})\subseteq E$ can be set to the weight of (*x*, *y*) in $(\vec {G},\sigma )$. However, since $U_2(\vec {G},{\mathcal {V}})$ and $U_3(\vec {G},{\mathcal {V}})$ contain elements that are not in *E*, one also would require weights for these “non-arcs”. Since, in the simplest case, “non-arcs” correspond to pairs of sequences that do not reach a certain similarity threshold (see e.g. [35]), the task of assigning weights to them does not seem trivial. In any case, whether incorporating such values of confidence is helpful needs to be assessed in practice.

The purpose of this contribution is to establish a sound theoretical foundation for practical approaches to BMG editing and to demonstrate that the problem can be solved for interestingly large instances at reasonable accuracy. In computational biology, however, much larger problems than the ones considered here would also be of interest. Less emphasis has been placed here on computational efficiency and scalability of different algorithmic variants. We leave this as topic for future research. Given the performance advantage of community detection over minimization of the *UR*-cost in each step, it seems most promising to focus on community detection methods that scale well for very large systems. The Louvain method seems to be a promising candidate, since it has been applied successfully to large networks in the past [28]. This is largely due to the fact that the change of modularity in response to moving a vertex between modules can be computed efficiently. We suspect that a comparably fast computation of the *UR*-cost may also be possible; this does not appear to be trivial, however. Moreover, the method could probably be accelerated by moving vertices into the community of the first neighbor such that this results in a (not necessarily optimal) improvement of the *UR*-cost. A similar randomization approach has already shown to only slightly affect the clustering quality in terms of modularity [37].

Since the restriction of a (be)BMG to a subset of colors is again a (be)BMG, it may also be possible to remove large parts of the noise by editing induced subgraph on a moderate number of colors, possibly using information of the phylogeny of the species to select species (= color) sets. Presumably, color sets with sufficient overlaps will need to be considered. A systematic analysis of this idea, however, depends on scalable BMG editing for large instances and goes beyond the scope of this contribution.

A potential shortcoming of the empirical analysis above is the simplistic error model, i.e., the independent perturbation of arcs (and non-arcs). Better models will depend on the investigation of BMGs derived from real-life sequence data. Such data is often burdened with systematic errors arising e.g. from the fact that a common ancestry often cannot be detected for very large evolutionary distances and from unequal mutation rates during the evolution of gene families, see e.g. [5, 38, 39] for more in-depth discussions of these issues. Benchmarking using real-life data, however, is a difficult task because the ground truth is unknown and large, well-curated data sets are not available. Our results so far suggest that a good performance w.r.t. the input digraph is also an indicator for a good performance w.r.t. the true digraph (cf. Figs. 7 and 8, green vs. blue boxplots). Moreover, they at least suggest that realistic BMG data can be processed with sufficient accuracy and efficiency to make BMGs an attractive alternative to classical phylogenetic methods. We indeed obtained promising results in a first application of our editing heuristics to the protein-coding genes of eleven Aquificales species. The construction of bioinformatics workflows to process best hit data, e.g. at the first processing stage of ProteinOrtho [35], is a logical next step.

## Figures and Tables

**Fig. 14:**
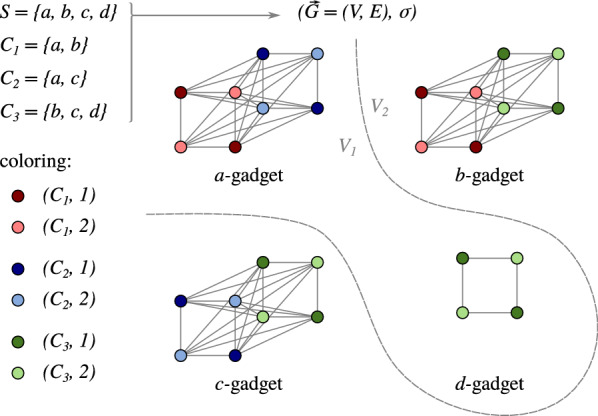
Example for the reduction from an instance $({\mathfrak {C}},S)$ of Set Splitting to an instance $(\vec {G},\sigma )$ with $k=0$ of BPURC, as specified in the proof of Thm. 30. In this example, we have $S=\{a,b,c,d\}$ and ${\mathfrak {C}}=\{C_1,C_2,C_3\}$. By construction, all arcs are bidirectional and thus, arrow heads are omitted in the drawing of $(\vec {G},\sigma )$. A solution for $({\mathfrak {C}},S)$ is $S_1=\{a,d\}$ and $S_2=\{b,c\}$. The latter is equivalent to a solution of BPURC by “separating” the *a*- and *d*-gadget from the *b*- and *c*-gadget as indicated by the dashed line. The latter yields a bipartition ${\mathcal {V}}=\{V_1,V_2\}$ of $V(\vec {G})$ that solves BPURC with input $(\vec {G},\sigma ,k=0)$. Note, slight changes of the input $({\mathfrak {C}},S)$ to $S'=S\setminus \{d\}$ and ${\mathfrak {C}}'=\{C_1,C_2,C_3\setminus \{d\}\}$ would yield an instance of Set Splitting that has no yes-answer. In this case, the *d*-gadget would disappear from $(\vec {G},\sigma )$ resulting in the digraph $(\vec {G}',\sigma ')$. It is easy to see that there is no bipartition ${\mathcal {V}}=\{V_1,V_2\}$ of $V(\vec {G}')$ such that $\sigma (V_1)=\sigma (V_2)=\sigma (V(\vec {G}'))$ and no gadget gets split up between $V_1$ and $V_2$; two necessary properties to obtain a solution for BPURC with input $(\vec {G}',\sigma ')$ and $k=0$ (cf. proof of Thm. 30)

**Fig. 15:**
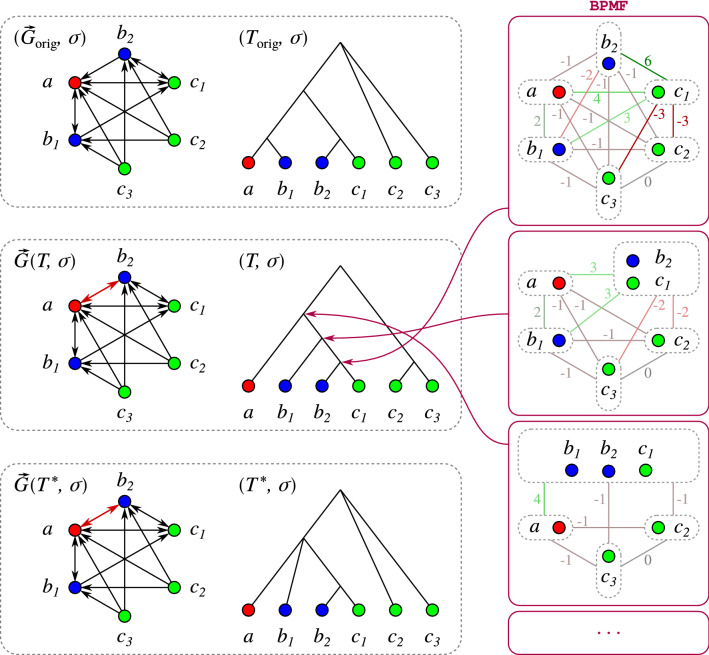
Example showing that BPMF is not a consistent heuristic for MaxRTC, and that Alg. 1 with BPMF is not a consistent heuristic for BMG editing. See the text for a detailed description

**Fig. 16:**
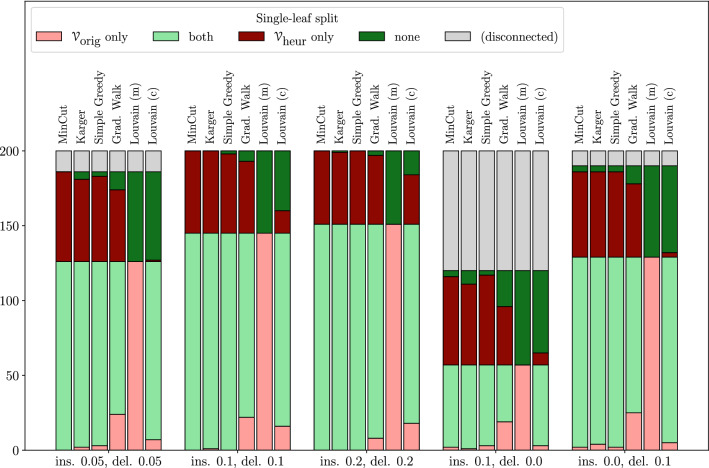
Abundance of single-leaf splits for pairs of BMGs $(\vec {G}_{\text {orig}},\sigma )$ and disturbed digraphs $(\vec {G},\sigma )$ (both with vertex set *V*). The partition ${\mathcal {V}}_{\text {orig}}$ corresponds to the connected components of the Aho graph $H_\text {orig}{:}{=}[{\mathcal {R}}(\vec {G}_{\text {orig}},\sigma ), V]$ and, hence, to the partition induced by the subtrees of the children of the root of the LRT $(T,\sigma )$ of $(\vec {G}_{\text {orig}},\sigma )$ (cf. Prop. 10). The partition ${\mathcal {V}}_\text {heur}$ corresponds to the partition of *V* as determined by one of the partitioning methods (based on $H{:}{=}[{\mathcal {R}}(\vec {G},\sigma ), V]$). The gray parts of the bars comprise those instances for which *H* is disconnected. The light and dark red bars indicate the amount of digraphs for which only ${\mathcal {V}}_{\text {orig}}$ or ${\mathcal {V}}_\text {heur}$, resp., is a single-leaf split, while light and dark green bars represent instances for which both and none of the two partitions, resp., are single-leaf splits. Note that the partitions were not compared explicitly, in particular, the identified singletons in ${\mathcal {V}}_\text {heur}$ in the light green instances may deviate from those in ${\mathcal {V}}_{\text {orig}}$ in some cases. Example plot for $|V|=30$ vertices and $|\sigma (V)|=10$ colors in each digraph. 200 generated digraph pairs per combination of arc insertion (ins.) and deletion (del.) probabilities

## Data Availability

Software implementing the algorithms described in this contribution is available in the Python package bmg-edit at https://github.com/david-schaller/bmg-edit. The simulation was performed using the library AsymmeTree [5] (https://github.com/david-schaller/asymmetree). The scripts used for simulation and comparison of the algorithms, the protein sequences of the eleven Aquificales species, and the results from which the plots were created are available at https://www.bioinf.uni-leipzig.de/supplements/21-003.

## Appendix

### Proofs

#### Proof of Lemma 13

##### Lemma 13

13*Let*$(\vec {G}=(V,E),\sigma )$*be a properly vertex-colored digraph and let*${\mathcal {V}}=\{V_1,\dots ,V_k\}$*be a partition of**V**with*$|{\mathcal {V}}|=k\ge 2$. *Then*

##### *Proof*

We first note that $U_1{:}{=}U_1(\vec {G},{\mathcal {V}})$, $U_2{:}{=}U_2(\vec {G},{\mathcal {V}})$ and $U_3{:}{=}U_3(\vec {G},{\mathcal {V}})$ are pairwise disjoint. Furthermore, we have $x\ne y$ and $\sigma (x)\ne \sigma (y)$ for every  and every $(x,y)\in U(\vec {G},{\mathcal {V}})$. Moreover, recall that ${\mathcal {T}}({\mathcal {V}})$ is the set of trees *T* on *V* that satisfy ${\mathcal {V}} = \{L(T(v)) \mid v\in \mathsf {child}_{T}(\rho _T) \}$. Therefore, there is a one-to-one correspondence between the $k\ge 2$ sets in ${\mathcal {V}}$ and the children $\mathsf {child}_{T}(\rho _{T})$ of the root $\rho _{T}$ for any $T\in {\mathcal {T}}({\mathcal {V}})$. We denote by $v_{i}$ the child corresponding to $V_{i}\in {\mathcal {V}}$; thus $V_{i}=L(T(v_{i}))$.

We first show that  implies $(x,y)\in U(\vec {G},{\mathcal {V}})$. Let $T\in {\mathcal {T}}({\mathcal {V}})$ be chosen arbitrarily, and let $\rho$ be its root. Suppose that $(x,y)\in U_1$. Thus, we have $(x,y)\in E$, $\sigma (y)\in \sigma (L(T(v_{i})))$, $x\preceq _{T} v_{i}$ and $y\preceq _{T} v'$ for some $v'\in \mathsf {child}_{T}(\rho )\setminus \{v_{i}\}$. Moreover, $\sigma (y)\in \sigma (L(T(v_{i})))$ implies that there is a vertex $y'\preceq _{T}v_{i}$ with $\sigma (y')=\sigma (y)$. Taken together, we obtain ${{\,\mathrm{lca}\,}}_{T}(x,y')\preceq _T v_{i} \prec _T \rho = {{\,\mathrm{lca}\,}}_{T}(x,y)$, and thus $(x,y)\notin E(\vec {G}(T,\sigma ))$. If $(x,y)\in U_2$, we have $(x,y)\notin E$, $\sigma (y)\notin \sigma (L(T(v_{i})))$, $x\preceq _{T}v_{i}$ and $y\preceq _{T} v'$ for some $v'\in \mathsf {child}_{T}(\rho )\setminus \{v_{i}\}$. Moreover, $\sigma (y)\notin \sigma (L(T(v_{i})))$ implies that there is no vertex $y'\preceq _{T}v_{i}$ with $\sigma (y')=\sigma (y)$. Thus, ${{\,\mathrm{lca}\,}}_{T}(x,y') = {{\,\mathrm{lca}\,}}_{T}(x,y)= \rho$ holds for all $y'$ of color $\sigma (y')=\sigma (y)$, and thus $(x,y)\in E(\vec {G}(T,\sigma ))$. Finally, suppose $(x,y)\in U_3$. We have $(x,y)\notin E$ and *y* is the only leaf of its color in $L(T(v_{i}))$. Therefore, there is no vertex $y'$ with $\sigma (y')=\sigma (y)$ and ${{\,\mathrm{lca}\,}}_{T}(x,y') \prec _{T} {{\,\mathrm{lca}\,}}_{T}(x,y)$, and thus $(x,y)\in E(\vec {G}(T,\sigma ))$. In summary, one of the conditions in Def. 12 is satisfied for *T* in all three cases. Since *T* was chosen arbitrarily, we conclude

In order to show that , we distinguish *Case (a)*: $(x,y)\in E$ and $(x,y)\notin E(\vec {G}(T,\sigma ))$ holds for all $T\in {\mathcal {T}}({\mathcal {V}})$, and *Case (b)*: $(x,y)\notin E$ and $(x,y)\in E(\vec {G}(T,\sigma ))$ holds for all $T\in {\mathcal {T}}({\mathcal {V}})$.

*Case (a).*$(x,y)\in E$ implies $\sigma (x)\ne \sigma (y)$. Moreover, there is a vertex $y'$ with $\sigma (y')=\sigma (y)$, and ${{\,\mathrm{lca}\,}}_{T}(x,y')\prec _{T}{{\,\mathrm{lca}\,}}_{T}(x,y)$ for every $T\in {\mathcal {T}}({\mathcal {V}})$ because $(x,y)\notin E(\vec {G}(T,\sigma ))$. Since this is true for all trees in ${\mathcal {T}}({\mathcal {V}})$, there must be a set $V_{i}\in {\mathcal {V}}$ such that $x, y'\in V_{i}$, and in particular $\sigma (y')=\sigma (y)\in \sigma (V_{i})$. Now suppose, for contradiction, that $y\in V_{i}$ and thus $x,y,y'\in V_{i}$. In this case, we can choose a tree $T\in {\mathcal {T}}({\mathcal {V}})$ such that $x,y\prec _{T}v_{i}$ for some child $v_{i}\in \mathsf {child}_{T}(\rho _T)$ and ${{\,\mathrm{lca}\,}}_{T}(x,y)\preceq {{\,\mathrm{lca}\,}}_{T}(x,y')$ hold for all $y'$ of color $\sigma (y')=\sigma (y)$. Hence, we obtain $(x,y)\in E(\vec {G}(T,\sigma ))$ for this tree; a contradiction. Therefore, we conclude that $y\in V\setminus V_{i}$. In summary, all conditions for $U_1$ are satisfied, and thus $(x,y)\in U_1$.

*Case (b).* We have $(x,y)\notin E$ and $(x,y)\in E(\vec {G}(T,\sigma ))$ for all $T\in {\mathcal {T}}({\mathcal {V}})$. Let $V_{i}\in {\mathcal {V}}$ such that $x\in V_{i}$. We distinguish the two cases (i) $y\notin V_{i}$, and (ii) $y\in V_{i}$. In Case (i), suppose, for contradiction, that $\sigma (y)\in \sigma (V_{i})$. Then, for every tree $T\in {\mathcal {T}}({\mathcal {V}})$, there must be a vertex $y'$ of color $\sigma (y)$ such that ${{\,\mathrm{lca}\,}}_{T}(x,y')\preceq _{T} v_{i}\prec _{T}\rho _T={{\,\mathrm{lca}\,}}_{T}(x,y)$, contradicting $(x,y)\in E(\vec {G}(T,\sigma ))$. Therefore, we conclude $\sigma (y)\notin \sigma (V_{i})$. It follows that $(x,y)\in U_2$. In Case (ii), assume, for contradiction, that there is a vertex $y'\in V_{i}$ of color $\sigma (y)$ such that $y\ne y'$. This together with $\sigma (y')=\sigma (y)\ne \sigma (x)$ implies that all three vertices $x,y,y'$ are pairwise distinct. Since in addition $x,y,y'\in V_{i}$, we can choose a tree $T\in {\mathcal {T}}({\mathcal {V}})$ such that $x,y,y'\prec _{T}v_{i}$ for some child $v_{i}\in \mathsf {child}_{T}(\rho _T)$ and ${{\,\mathrm{lca}\,}}_{T}(x,y')\prec _{T}{{\,\mathrm{lca}\,}}_{T}(x,y)$; a contradiction to $(x,y)\in E(\vec {G}(T,\sigma ))$ for all $T\in {\mathcal {T}}({\mathcal {V}})$. Therefore, we conclude that *y* is the only vertex of its color in $V_{i}$. It follows that $(x,y)\in U_3$. $\square$

#### Proof of Lemma 15

In order to prove Lemma 15, we first need the following technical result which shows that the editing of an arc in Alg. 2 will not be reversed in the subsequent recursion step.

##### Lemma 31

*Let*$(\vec {G}=(V,E),\sigma )$*be a properly vertex-colored digraph,*${\mathcal {V}}=\{V_1,\dots ,V_k\}$*a partition of**V**with*$|{\mathcal {V}}|=k\ge 2$, *and*${\mathcal {V}}_i=\{V_{i,1},\dots ,V_{i,l}\}$, $1\le i\le k$, *a partition of*$V_i$*with*$|{\mathcal {V}}_i|=l\ge 2$. *Moreover, let*$(\vec {G}'{:}{=}\vec {G}\triangle U(\vec {G},{\mathcal {V}}),\sigma )$*be the colored digraph that is obtained by applying the edits in*$U(\vec {G},{\mathcal {V}})$*to*$(\vec {G},\sigma )$. *Then*$U(\vec {G},{\mathcal {V}})\cap U(\vec {G}'[V_i],{\mathcal {V}}_i)=\emptyset$.

##### *Proof*

Let $\vec {G}'_i{:}{=}\vec {G}'[V_i]$. The sets of unsatisfiable relations $U(\vec {G},{\mathcal {V}})$ and $U(\vec {G}'_i, {\mathcal {V}}_i)$ are given by the (disjoint) unions  and , respectively (cf. Lemma 13). First, let $(x,y)\in U_1(\vec {G},{\mathcal {V}})$. Since, by definition of $U_1(\vec {G},{\mathcal {V}})$, *x* and *y* are contained in different sets of the partition ${\mathcal {V}}$, they cannot be both contained in $V_i$ and thus, $U_1(\vec {G},{\mathcal {V}}) \cap U(\vec {G}'_i, {\mathcal {V}}_i)=\emptyset$. One analogously argues that $U_2(\vec {G},{\mathcal {V}}) \cap U(\vec {G}'_i, {\mathcal {V}}_i)=\emptyset$. Now, assume for contradiction that $(x,y)\in U_3(\vec {G},{\mathcal {V}})\cap U(\vec {G}'_i, {\mathcal {V}}_i)$. By definition of $U(\vec {G}'_i, {\mathcal {V}}_i)$, this implies $x,y\in V_i$. Moreover, by definition of $U_3(\vec {G},{\mathcal {V}})$, we have $(x,y)\notin E$, which immediately implies $(x,y)\in E(\vec {G}'_i)$. By Lemma 13, we therefore conclude $(x,y)\in U_1(\vec {G}'_i,{\mathcal {V}}_i)$. Let $V_{i,j}$ be the set of the partition ${\mathcal {V}}_i$ which contains *x*. Then, by definition of $U_1(\vec {G}'_i,{\mathcal {V}}_i)$, the color of *y* is contained in both $V_{i,j}$ and $V_i\setminus V_{i,j}$, i.e., $V_i$ contains at least two vertices of color $\sigma (y)$. However, $(x,y)\in U_3(\vec {G},{\mathcal {V}})$ and $y\in V_i$ together imply that *y* is the only vertex of its color in $V_i$; a contradiction. $\square$

We are now in the position to prove the more general

##### Lemma 15

15*All edit sets*$U(\vec {G}^*[V'],{\mathcal {V}})$*constructed in Alg*. 2 *are pairwise disjoint.*

##### *Proof*

First note that, by Lemma 13, we have $\sigma (x)\ne \sigma (y)$ for all $(x,y)\in U(\vec {G}^*[V'], {\mathcal {V}})$. Hence, the digraph $(\vec {G}^*,\sigma )$ remains properly colored during the whole recursion. Moreover, recursive calls on a set $V'$ with $|V'|=1$ trivially contribute with a *UR*-cost of zero.

By construction, the partitions in consecutive calls of Edit() form a hierarchical refinement such that in each recursive call a single element of $V_i\in {\mathcal {V}}$ is refined. Clearly edit sets encountered in independent branches of the recursion tree are disjoint because they pertain to disjoint vertex sets. For directly consecutive calls of Edit(), Lemma 31 states that the edits sets are disjoint. Now consider two recursive call on $V'$ and $V''$ with $V''\subset V'$ that are not directly consecutive. Let ${\mathcal {V}}'$ and ${\mathcal {V}}''$, resp., be the partitions chosen for the vertex sets $V'$ and $V''$ of $\vec {G}'$ and $\vec {G}''$ at the beginning of the two recursion steps. We can apply the same arguments as in the proof of Lemma 31 to conclude that $U_i(\vec {G}'[V'],{\mathcal {V}}') \cap U(\vec {G}''[V''],{\mathcal {V}}'')=\emptyset$, $i\in \{1,2\}$. Finally, assume, for contradiction, that $(x,y)\in U_3(\vec {G}'[V'],{\mathcal {V}}') \cap U(\vec {G}''[V''],{\mathcal {V}}'')$. By definition of $U(\vec {G}''[V''], {\mathcal {V}}'')$, this implies $x,y\in V''$. Moreover, by definition of $U_3(\vec {G}'[V'],{\mathcal {V}}')$, we have $(x,y)\notin E(\vec {G}')$, which immediately implies $(x,y)\in E(\vec {G}'\triangle U(\vec {G}'[V'],{\mathcal {V}}'))$, i.e., (*x*, *y*) is an arc after the editing in this step. Since both *x*, *y* are contained in $V''$, it follows from Lemma 13 that all edit steps on the way from $\vec {G}'[V'']$ to $\vec {G}''[V'']$ must be performed by the set $U_3$, i.e., they exclusively correspond to arc insertions. Therefore, (*x*, *y*) is still an arc in $\vec {G}''[V'']$. By Lemma 13, we therefore conclude that $(x,y)\in U_1(\vec {G}''[V''],{\mathcal {V}}'')$. Let $V_{x}$ be the set of the partition ${\mathcal {V}}''$ that contains *x*. Then, by definition of $U_1(\vec {G}''[V''],{\mathcal {V}}'')$, the color of *y* is contained in both $V_{x}$ and $V''\setminus V_{x}$, i.e., $V''$ contains at least two vertices of color $\sigma (y)$. However, $(x,y)\in U_3(\vec {G}'[V'],{\mathcal {V}}')$ and $y\in V''\subset V_{x,y}$ for some $V_{x,y}\in {\mathcal {V}}'$ together imply that *y* is the only vertex of its color in $V''$; a contradiction. $\square$

#### Proof of Theorem 30

In this section, we show that (B)PURC is NP-hard by reduction from Set Splitting.

##### Problem 4

(Set Splitting)

**Table:**

*Input:*	A collection ${\mathfrak {C}}$ of subsets of a finite set*S*,
	denoted by $({\mathfrak {C}},S)$.
*Question:*	Is there a bipartition of *S* into two subsets
	$S_1$ and $S_2$ such that no subset in ${\mathfrak {C}}$ is
	entirely contained in either $S_1$ or $S_2$?

##### Proposition 32

[40] Set Splitting *is NP-complete.*

##### Theorem 30

BPURC *is NP-complete.*

##### *Proof*

Given a properly colored digraph $(\vec {G}=(V,E),\sigma )$ and a bipartition ${\mathcal {V}}$ of *V*, the set $U(\vec {G},{\mathcal {V}})$ and thus the *UR*-cost $c(\vec {G},{\mathcal {V}})=|U(\vec {G},{\mathcal {V}})|$ can be computed in polynomial time according to Cor. 14. Therefore, BPURC is contained in NP. To show NP-hardness, we use reduction from Set Splitting.

Let $({\mathfrak {C}},S)$ be an instance of Set Splitting. We may assume w.l.o.g. that $|C|\ge 2$ holds for all $C\in {\mathfrak {C}}$, since otherwise there is no solution at all for Set Splitting. In addition, we assume that $\bigcup _{C\in {\mathfrak {C}}}C = S$. To see that this does not yield a loss of generality, suppose that $\bigcup _{C\in {\mathfrak {C}}}C =S' \subsetneq S$. If $\{S'_1,S'_2\}$ is a solution for $({\mathfrak {C}},S')$ then no subset in ${\mathfrak {C}}$ is entirely contained in either $S'_1$ or $S'_2$. Therefore, we can construct a solution $(S_1,S_2)$ for $({\mathfrak {C}},S)$ by arbitrarily adding the elements in $S\setminus S'$ to either $S'_1$ or $S'_2$. In contrast, $\{S_1\cap S',S_2\cap S'\}$ is a solution for $({\mathfrak {C}},S')$ provided that $\{S_1,S_2\}$ is a solution for $({\mathfrak {C}},S)$.

Now, let $({\mathfrak {C}},S)$ be an instance of Set Splitting and define, for all $s\in S$, the set ${\mathfrak {C}}(s) {:}{=}\{C\mid C\in {\mathfrak {C}}, s\in C\}$ as the subset of ${\mathfrak {C}}$ that comprises all elements $C\in {\mathfrak {C}}$ that contain *s*. Note that ${\mathfrak {C}}(s) \ne \emptyset$ for all $s\in S$, since we have assumed $\bigcup _{C\in {\mathfrak {C}}}C = S$, i.e., every $s\in S$ is contained in some element of ${\mathfrak {C}}$.

We construct a digraph $(\vec {G}=(V,E),\sigma )$ that serves as input for BPURC as follows:

*Step 1:*: For all $s\in S$, construct an *s*-gadget ${\mathcal {G}}_s$ as follows: (i): For all $C\in {\mathfrak {C}}(s)$, add four new vertices to ${\mathcal {G}}_s$ of which two are colored with (*C*, 1) and the other two with (*C*, 2). (ii): Add arcs (*x*, *y*), (*y*, *x*) between all $x,y\in V({\mathcal {G}}_s)$ with $\sigma (x)\ne \sigma (y)$.
*Step 2:*: Set , and preserve the coloring of the vertices within the *s*-gadgets to obtain the digraph $(\vec {G},\sigma )$.

By construction, we have $|V({\mathcal {G}}_s)| = 4|{\mathfrak {C}}(s)|$ and $|V| = \sum _{s\in S}\; 4|{\mathfrak {C}}(s)|\le 4|{\mathfrak {C}}||S|$. Hence, the construction of $(\vec {G},\sigma )$ can be achieved in polynomial time. Moreover, by construction, $\{V({\mathcal {G}}_s)\mid s\in S\}$ forms a partition of *V* and there are no arcs between vertices of distinct *s*-gadgets. Furthermore, $\sigma (V) = {\mathfrak {C}}\times \{1,2\}$. An illustrative example of such a constructed digraph $(\vec {G},\sigma )$ is provided in Fig. 14.

We continue by showing that an instance $({\mathfrak {C}},S)$ of Set Splitting has a yes-answer if and only if BPURC has a yes-answer for the input digraph $(\vec {G}=(V,E),\sigma )$ constructed above and $k=0$. In particular, we will show that $\{S_1,S_2\}$ is a solution of $({\mathfrak {C}},S)$ if and only if ${\mathcal {V}} = \{V_1,V_2\}$ with $V_i=\cup _{s\in S_i} V({\mathcal {G}}_s)$, $i\in \{1,2\}$ is a solution for $(\vec {G},\sigma )$ where $c(\vec {G},{\mathcal {V}}) = 0$.

Recall that the set of unsatisfiable relations $U(\vec {G},{\mathcal {V}})$ of a bipartition ${\mathcal {V}}$ of *V* is given by the (disjoint) union  of the three sets $U_1 {:}{=}U_1(\vec {G},{\mathcal {V}})$, $U_2 {:}{=}U_2(\vec {G},{\mathcal {V}})$ and $U_3 {:}{=}U_3(\vec {G},{\mathcal {V}})$ (cf. Lemma 13).

First suppose that Set Splitting with input $({\mathfrak {C}},S)$ has a yes-answer and let $\{S_1,S_2\}$ be one of its solutions. Hence, no subset in ${\mathfrak {C}}$ is entirely contained in either $S_1$ or $S_2$, and both sets must be non-empty. Consider the set ${\mathcal {V}} = \{V_1,V_2\}$ with $V_i=\cup _{s\in S_i}\, V({\mathcal {G}}_s)$, $i\in \{1,2\}$. Since $\{S_1,S_2\}$ is a bipartition of *S* and $\{V({\mathcal {G}}_s)\mid s\in S\}$ is a partition of *V*, we conclude that ${\mathcal {V}}$ is a bipartition of *V* and that $V({\mathcal {G}}_s)$ is entirely contained in either $V_1$ or $V_2$ for all $s\in S$. Together with the fact that there are no arcs in $\vec {G}$ between vertices of distinct *s*-gadgets this implies that $U_1 =\emptyset$.

In order to verify that $U_2 = U_3 = \emptyset$, we first show that $\sigma (V_1)=\sigma (V_2) = \sigma (V)$ and that $V_1$ and $V_2$ contain at least two vertices of every color, respectively. Consider two arbitrary pairs $(C,1), (C,2)\in \sigma (V)={\mathfrak {C}}\times \{1,2\}$. Since $\{S_1,S_2\}$ is a solution for Set Splitting with input $({\mathfrak {C}},S)$, there are vertices $s\in C\cap S_1$ and $s'\in C\cap S_2$ and thus, $V({\mathcal {G}}_s)\subseteq V_1$ and $V({\mathcal {G}}_{s'})\subseteq V_2$. By construction, each of the sets $V({\mathcal {G}}_s)$ and $V({\mathcal {G}}_{s'})$ contains two vertices of color (*C*, 1) and two vertices of color (*C*, 2). Since $V({\mathcal {G}}_s)\subseteq V_1$ and $V({\mathcal {G}}_{s'})\subseteq V_2$, the sets $V_1$ and $V_2$ each contain two vertices of both colors (*C*, 1) and (*C*, 2). Since $(C,1), (C,2)\in \sigma (V)$ are arbitrary and $\sigma (V)= {\mathfrak {C}}\times \{1,2\}$, we can conclude that $\sigma (V_1)=\sigma (V_2) = \sigma (V)$, and that $V_1$ and $V_2$ contain at least two vertices of every color. Now, $\sigma (V_1)=\sigma (V_2)$ implies that $U_2=\emptyset$. Moreover, since $V_1$ and $V_2$ contain at least two vertices of every color, we also have that $U_3=\emptyset$. In summary, we have , and thus, $c(\vec {G},{\mathcal {V}})=0$. Therefore, BPURC with input $(\vec {G},\sigma ,k=0)$ has a yes-answer.

Now suppose BPURC with input $(\vec {G},\sigma ,k=0)$ has a yes-answer and thus, a solution ${\mathcal {V}}=\{V_1,V_2\}$. Consequently, . We first show that both $V_1$ and $V_2$ must contain a vertex of every color in $\sigma (V) = {\mathfrak {C}}\times \{1,2\}$. To this end, we assume for contradiction that w.l.o.g. $V_1$ contains no vertex of color (*C*, 1) for some $C\in {\mathfrak {C}}$. Since $|C|\ge 2$, *C* contains two distinct elements $s, s'\in S$. Note that $C\in {\mathfrak {C}}(s)$ and $C\in {\mathfrak {C}}(s')$. By construction in Step 1, there are vertices $y\in V({\mathcal {G}}_s)$ and $y'\in V({\mathcal {G}}_{s}')$ of color $\sigma (y)=\sigma (y')=(C,1)$. Since $(C,1)\notin \sigma (V_1)$, it must hold that $y,y'\in V_2$. Now consider an arbitrary vertex $x\in V_1$. Note that $(C,1)\notin \sigma (V_1)$ implies $\sigma (x)\ne (C,1)$. Since ${\mathcal {G}}_s$ and ${\mathcal {G}}_{s'}$ are, by construction, vertex disjoint, *x* cannot belong two both gadgets ${\mathcal {G}}_s$ and ${\mathcal {G}}_{s'}$. Therefore, we can choose ${\tilde{y}}\in \{y,y'\}$ such that *x* and ${\tilde{y}}$ belong to distinct gadgets, and we obtain $(x,{\tilde{y}})\notin E$ by construction. This together with $x\in V_1$, ${\tilde{y}}\in V_2=V\setminus V_1$ and $\sigma ({\tilde{y}})=(C,1)\notin \sigma (V_1)$ implies $(x,{\tilde{y}})\in U_2$. Hence, $U_2\ne \emptyset$; a contradiction. Therefore, we conclude that both $V_1$ and $V_2$ contain vertices of all colors in $\sigma (V)={\mathfrak {C}}\times \{1,2\}$.

We continue by showing that $V({\mathcal {G}}_s)$ is entirely contained in either $V_1$ or $V_2$ for all $s\in S$. To this end, assume for contradiction that there is a gadget ${\mathcal {G}}_s$ such that $W_1{:}{=}V_1\cap V({\mathcal {G}}_s)$ and $W_2{:}{=}V_2\cap V({\mathcal {G}}_s)$ are both non-empty. Since $V({\mathcal {G}}_s)$ forms a connected component in $(\vec {G},\sigma )$ and all arcs are bidirectional by construction, we can find two vertices $x\in W_1$ and $y\in W_2$ such that $(x,y)\in E$. This together with the facts that *x* and *y* are in distinct sets $V_1$ and $V_2$ and that both $V_1$ and $V_2$ contain all colors of $\sigma (V)$, implies that $(x,y)\in U_1$. Hence, $U_1\ne \emptyset$; a contradiction. Therefore, the vertex set of each *s*-gadget is entirely contained in either $V_1$ or $V_2$.

We can construct a well-defined partition $\{S_1,S_2\}$ of *S* such that $s\in S_i$ if and only if $V({\mathcal {G}}_s)\subseteq V_{i}$, $i\in \{1,2\}$. By construction, there are vertices of color (*C*, 1) and (*C*, 2) in ${\mathcal {G}}_s$ if and only if $s\in C$. This together with the fact that both $V_1$ and $V_2$ contain vertices of all colors ${\mathfrak {C}}\times \{1,2\}$ implies that $S_1\cap C$ and $S_2\cap C$ are both non-empty for every $C\in {\mathfrak {C}}$. Hence, $\{S_1, S_2\}$ is a solution for Set Splitting with input $({\mathfrak {C}},S)$. $\square$

### B BPMF is not a consistent heuristic for MaxRTC

The example in Fig. 15 shows that Alg. 1 in combination with the Best-Pair-Merge-First (BPMF) heuristic [16, 18] is not a consistent heuristic for BMG editing. In particular, BPMF is not consistent for MaxRTC. The input digraph $(\vec {G}_\text {orig}=(V,E),\sigma )$ is a BMG and explained by $(T_\text {orig},\sigma )$. Therefore, its set of informative triples

$$\begin{aligned} {} {\mathcal {R}}{:}{=}{\mathcal {R}}(\vec {G}_\text {orig},\sigma ) = &\{ ab_1|b_2,\, ac_1|c_2,\, ac_1|c_3,\\& b_1c_1|c_2,\, b_1c_1|c_3,\, b_2c_1|c_2,\\& b_2c_1|c_3,\, c_1b_2|b_1\} \end{aligned}$$

is consistent (cf. Prop. 10). On the right-hand side of Fig. 15, the first three cluster merging steps in BPMF with input ${\mathcal {R}}$ are shown where the numbers are the scores $score(S_i, S_j)$ for each pair of clusters $S_i$ and $S_j$ as defined in [16]. The pink arrows link inner vertices of the resulting binary tree $(T,\sigma )$ and the corresponding cluster merging step based on the maximal score. The tree $(T,\sigma )$ does not display the triple $ab_1|b_2$. As a consequence, its BMG $\vec {G}(T,\sigma )$ contains the additional arc $(a,b_2)$, and the triple set ${\mathcal {R}}^*$ extracted from *T* in Alg. 1 is a proper subset of ${\mathcal {R}}$. In particular, the final editing result $\vec {G}(T^*,\sigma )$ with $T^*={{\,\mathrm{Aho}\,}}({\mathcal {R}}^*,V)$ also contains the arc $(a,b_2)$ which was not present in the original BMG.

### C Analysis of single-leaf splits

Fig. 16 quantifies the abundance of single-leaf splits on the same instances as in Fig. 6. We distinguish between single-leaf splits that are correct w.r.t. the Aho graph $H_\text {orig}$ of the original unperturbed digraph, and single-leaf splits that are not present in the unperturbed target. MinCut, Karger, Simple Greedy and Gradient Walk frequently produce single-leaf splits that are not present in $H_\text {orig}$. The modularity-based Louvain method, in contrast, never returned a single-leaf split, even if it was present in $H_\text {orig}$. The modified Louvain method is most often in good agreement with $H_\text {orig}$ as far as single-leaf splits are concerned, at least for perturbation levels of 10% of insertions and deletions.
